# Clinical practice guidelines for IgG4‐related sclerosing cholangitis

**DOI:** 10.1002/jhbp.596

**Published:** 2019-01-18

**Authors:** Terumi Kamisawa, Takahiro Nakazawa, Susumu Tazuma, Yoh Zen, Atsushi Tanaka, Hirotaka Ohara, Takashi Muraki, Kazuo Inui, Dai Inoue, Takayoshi Nishino, Itaru Naitoh, Takao Itoi, Kenji Notohara, Atsushi Kanno, Kensuke Kubota, Kenji Hirano, Hiroyuki Isayama, Kyoko Shimizu, Toshio Tsuyuguchi, Tooru Shimosegawa, Shigeyuki Kawa, Tsutomu Chiba, Kazuichi Okazaki, Hajime Takikawa, Wataru Kimura, Michiaki Unno, Masahiro Yoshida

**Affiliations:** ^1^ Department of Internal Medicine, Tokyo Metropolitan Komagome Hospital Tokyo Japan; ^2^ Department of Gastroenterology Japanese Red Cross Nagoya Daini Hospital Nagoya Japan; ^3^ Department of General Internal Medicine Hiroshima University Graduate School of Biomedical & Health Science Hiroshima Japan; ^4^ Department of Diagnostic Pathology Kobe University Kobe Japan; ^5^ Department of Medicine Teikyo University School of Medicine Tokyo Japan; ^6^ Department of Community‐Based Medical Education Nagoya City University Graduate School of Medical Sciences Nagoya Japan; ^7^ Department of Medicine, Gastroenterology Shinshu University Matsumoto, Nagano Japan; ^8^ Department of Gastroenterology Second Teaching Hospital Fujita Health University Nagoya Japan; ^9^ Department of Radiology Kanazawa University Graduate School of Medical Sciences Kanazawa Japan; ^10^ Department of Gastroenterology Tokyo Womens’ Medical University Yachiyo Medical Center Yachiyo Japan; ^11^ Department of Gastroenterology and Metabolism Nagoya City University Graduate School of Medical Sciences Nagoya Japan; ^12^ Department of Gastroenterology and Hepatology Tokyo Medical University Tokyo Japan; ^13^ Department of Anatomic Pathology Kurashiki Central Hospital Kurashiki Japan; ^14^ Division of Gastroenterology Tohoku University Graduate School of Medicine Sendai Japan; ^15^ Department of Endoscopy Yokohama City University Hospital Yokohama Japan; ^16^ Department of Gastroenterology Tokyo Takanawa Hospital Tokyo Japan; ^17^ Department of Gastroenterology Graduate School of Medicine Juntendo University Tokyo Japan; ^18^ Department of Gastroenterology Tokyo Womens’ Medical University Tokyo Japan; ^19^ Endoscopy Center Chiba University Hospital Chiba Japan; ^20^ Division of Gastroenterology South‐Miyagi Medical Center Ohgawara Japan; ^21^ Department of Internal Medicine Matsumoto Dental University Matsumoto Japan; ^22^ Kansai Electric Power Hospital Osaka Japan; ^23^ The Third Department of Internal Medicine Division of Gastroenterology and Hepatology Kansai Medical University Moriguchi Japan; ^24^ Faculty of Medicine Departments of Gastroenterology and Gastroenterological, General, Breast, and Thyroid Surgery Yamagata University Yamagata Japan; ^25^ Division of Hepato‐Biliary Pancreatic Surgery Tohoku University Graduate School, of Medicine Sendai Japan; ^26^ Department of Hepato‐Biliary‐Pancreatic and Gastrointestinal Surgery School of Medicine International University of Health and Welfare Ichikawa Japan

**Keywords:** Bile duct, Guideline, IgG4, Sclerosing cholangitis, Steroid

## Abstract

IgG4‐related sclerosing cholangitis (IgG4‐SC) is a distinct type of cholangitis frequently associated with autoimmune pancreatitis and currently recognized as a biliary manifestation of IgG4‐related disease. Although clinical diagnostic criteria of IgG4‐SC were established in 2012, differential diagnosis from primary sclerosing cholangitis and cholangiocarcinoma is sometimes difficult. Furthermore, no practical guidelines for IgG4‐SC are available. Because the evidence level of most articles retrieved through searching the PubMed, Cochrane Library, and Igaku Chuo Zasshi databases was below C based on the systematic review evaluation system of clinical practice guidelines MINDS 2014, we developed consensus guidelines using the modified Delphi approach. Three committees (a guideline creating committee, an expert panelist committee for rating statements according to the modified Delphi method, and an evaluating committee) were organized. Eighteen clinical questions (CQs) with clinical statements were developed regarding diagnosis (14 CQs) and treatment (4 CQs). Recommendation levels for clinical statements were set using the modified Delphi approach. The guidelines explain methods for accurate diagnosis, and safe and appropriate treatment of IgG4‐SC.

## Introduction

IgG4‐related sclerosing cholangitis (IgG4‐SC) is a distinct type of cholangitis characterized by elevation of serum IgG4 levels, dense infiltration of IgG4‐positive plasma cells and lymphocytes, with fibrosis and obliterative phlebitis in the bile duct wall 1. IgG4‐SC, which is frequently associated with autoimmune pancreatitis (AIP), is currently recognized as a biliary manifestation of a systemic disorder termed IgG4‐related disease 2.

IgG4‐SC is diagnosed via a combination of imaging, serological, and histopathological findings, coexistence of other IgG4‐related diseases, and effectiveness of steroid therapy according to clinical diagnostic criteria of IgG4‐SC 2012 3. Cholangiographic findings of IgG4‐SC involving the hilar and/or intrahepatic bile ducts are similar to those of hilar cholangiocarcinoma or primary sclerosing cholangitis (PSC). Some IgG4‐SC cases were resected on suspicion of cholangiocarcinoma. PSC is a progressive and chronic disease with poor prognosis and does not respond to steroid therapy. Therefore, IgG4‐SC should be differentiated from the two diseases. However, no previous guidelines for IgG4‐SC have been published. The present guidelines describe methods for the accurate diagnosis, and safe and appropriate treatment of IgG4‐SC based on specialized gastroenterological knowledge, skills, and experience. Although gastroenterologists are the primary target users of these clinical practice guidelines, we cover a wide range of clinical issues for general clinicians, as various IgG4‐related diseases are frequently associated with IgG4‐SC. To understand characteristic features of IgG4‐SC, background questions for concept, etiology, epidemiology and prognosis were described. Adults are the primary patients addressed in the guidelines, and children are excluded.

The guidelines describe currently accepted standard management but do not force medical action. Appropriate management of patients should be determined based on the situation of each institute and patient.

## Methods

### Evidence level and establishing consensus guidelines using the Delphi approach

A total of 234 and 2,340 publications published between 1991 and March 2017 were retrieved from a search of the PubMed database using the key words “IgG4‐related sclerosing cholangitis” and “autoimmune pancreatitis”, respectively. There was only one randomized controlled trial report, and the evidence level of the other publications was below grade C (low quality) 4. Therefore, we developed consensus guidelines using the modified Delphi approach 5, 6. This method, which provides panelists with the opportunity to discuss their judgements between the ratings’ rounds, is suitable for the development of consensus guideline statement.

To establish the consensus guidelines, three committees were organized, as follows: a guideline‐creation committee for developing clinical questions (CQs) and statements (pancreatobiliary specialist [*n = *16], radiologist [*n = *1], and pathologist [*n = *2]), an expert panelist committee for rating statements according to the modified Delphi method (pancreatobiliary specialist [*n = *9] and pathologist [*n = *1]), and an evaluation committee (pancreatobiliary special physician [*n = *2], pancreatobiliary special surgeon [*n = *2], gastroenterologist [*n = *1], and guideline‐development specialist [*n = *1]) (Appendix 1). As there are only a few IgG4‐SC specialists, eight creation specialists doubled as expert panelists. However, these panelists did not assess items with which they were involved.

### Clinical question (CQ) preparation

The guideline‐creation committee proposed 18 CQs for diagnosis (14 CQs) and treatment (4 CQs) in consideration of PICO format, which described the population, intervention, control, and outcomes.

### Literature search and evidence assessment

Key words were extracted from the CQs, and academic papers were collected. A literature search was performed using the PubMed (MEDLINE), Cochrane Library, and Igaku Chuo Zasshi databases covering the period between 1995 and March 2017. Additionally, we searched the reference lists of articles identified by this search strategy and selected those that we judged to be relevant. Review articles and book chapters are cited to provide readers with more details and references. The search formula for each CQ will be available at the homepage of the Japan Biliary Association (http://www.tando.gr.jp/).

The guidelines were developed with the use of system of the clinical practice guidelines MINDS 2014 to evaluate strength of systematic reviews 4. The quality of evidence was graded as A (high), B (moderate), C (low), or D (very low).

### Process for developing the original draft

The guideline‐creation committee members developed, evaluated, and revised statements and comments for the CQs. The revised draft was also evaluated and revised through a review meeting of the guideline committee. The draft was discussed at a symposium and special session held on 29 September 2017, during the 53^rd^ Annual Meeting of the Japan Biliary Association.

### Establishing a consensus and grading the strength of recommendations using the modified Delphi method

Ratings for statements and comments pertaining to each CQ were developed using the modified Delphi approach. Ratings were on a 9‐point scale, with 1 being highly inappropriate and 9 being highly appropriate. A clinical statement receiving a median score greater than 7 from the panel was regarded as valid. The creating specialists revised some of the statements and comments after discussion with expert panelists. The revised statements and comments were then rated again. Based on the two‐round modified Delphi approach, final statements and comments were developed.

The strength of recommendations was classified as high (strong) (recommendation 1) or low (weak) (recommendation 2), as determined according to the following factors: quality of the evidence, patient's preferences, benefits and harms (risks), and cost. A recommendation was applied when more than 70% of the expert specialists (Delphi method) agreed. The use of “We recommend—” was adopted as the style for a strong recommendation. “We suggest—” was adopted as the style for a weak recommendation.

### Evaluation by the evaluating committee and public comments

The revised draft after the modified Delphi approach was evaluated by the evaluation committee using AGREE II 7. After final modifications were made, public comments were invited on the website of the Japan Biliary Association. Based on these public comments, some comments were modified.

### Background questions

#### What is IgG4‐SC?

- IgG4‐SC is SC induced via an autoimmune mechanism and responds dramatically to steroid therapy. Most cases are associated with systemic IgG4‐related diseases such as AIP.

*Comment:* IgG4‐SC is SC that responds dramatically to steroid therapy, with good clinical prognosis. Differential diagnosis from PSC, cholangiocarcinoma, pancreatic cancer, and secondary SC is necessary because these conditions show similar cholangiograms to IgG4‐SC. IgG4‐SC is a biliary manifestation of IgG4‐related diseases because it is associated with systemic IgG4‐related diseases such as AIP 8. The etiology is thought to involve an autoimmune mechanism because various antibodies are detected and the disease responds well to steroid therapy 9. IgG4‐SC is diagnosed according to clinical diagnostic criteria of IgG4‐SC 2012 3. It is treated by steroid therapy in the same manner as AIP.

Since the 1970s, cases of SC associated with chronic pancreatitis have been published overseas and in Japan. In most of these reports, pancreatic disease was diagnosed as chronic pancreatitis and biliary disease as PSC. Waldram et al. reported two SC cases associated with chronic pancreatitis, diabetes and Sjögren syndrome in 1975 10. Sjögren et al. reported two PSC cases that responded to steroid therapy 11.

In 1991, Kawaguchi et al. reported lymphoplasmacytic sclerosing pancreatitis with cholangitis as a variant of PSC in Japan by studying surgical specimens 12. Since 1996, some cases of SC meeting the diagnostic criteria of PSC but showing a better clinical course than classic PSC have been reported. These were reported as “atypical PSC” in order to discriminate them from classic PSC 13. The atypical PSC cases showed characteristic findings such as onset at older age, good response to steroid therapy, biliary drainage, no association with ulcerative colitis, and frequent association with characteristic chronic pancreatitis. After the concept of AIP was established, these cases have been reported as “sclerosing cholangitis with autoimmune pancreatitis” 14. After the concept of IgG4‐related diseases was established and isolated SC without AIP was reported, these cases have been reported as IgG4‐SC 15.

The term “IgG4‐associated sclerosing cholangitis” has also been used 16. “IgG4‐related sclerosing cholangitis” became the formal name at the 1^st^ International Symposium on IgG4‐related disease, with establishment of uniform international nomenclature and pathologic characteristics of IgG4‐related disease 17.

#### How is IgG4‐SC classified?

- Classifications based on cholangiograms and the association with AIP have been used.

Inclusion of type 1 IgG4‐SC into the IgG4‐SC category has been disputed.

**Figure 1:**
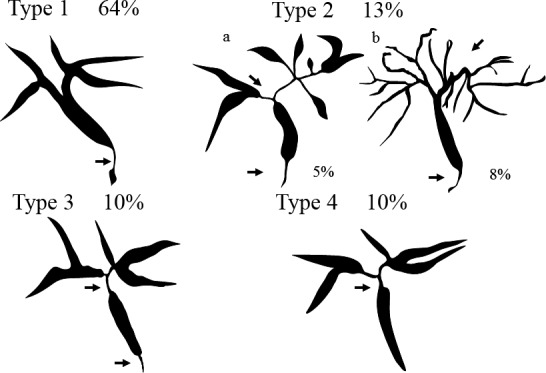
Cholangiographic classification of IgG4‐SC referred from Reference 18

*Comment*: Cholangiographic classification, which is useful for differential diagnosis, is the prevailing method 18 (Fig. 1). Type 1 IgG4‐SC involves stenosis only in the lower bile duct and thus should be differentiated from pancreatic cancer and cholangiocarcinoma 1. Type 2 IgG4‐SC, in which stenosis is diffusely distributed throughout the intrahepatic and extrahepatic bile ducts, should be differentiated from PSC and is further subdivided into two subtypes: type 2a, characterized by stricture of the intrahepatic bile ducts with prestenotic dilation; and type 2b, characterized by stricture of the intrahepatic bile ducts without prestenotic dilation and reduced bile duct branches, which should be discriminated from the “pruned‐tree” appearance in PSC. Type 3 IgG4‐SC is characterized by stenosis in the hilar hepatic lesions and lower bile duct. Type 4 IgG4‐SC presents with strictures of the bile duct only in the hilar hepatic lesions. The cholangiographic findings of types 3 and 4 IgG4‐SC should be discriminated from those of cholangiocarcinoma. A national survey performed in 2015 reported that the frequencies of the types of IgG4‐SC are 64% (type 1), 5% (type 2a), 8% (type 2b), 10% (type 3), and 10% (type 4) 19. Some type 2 cases can be differentiated from PSC by endoscopic retrograde cholangiography (ERC) findings but not differentiated from cholangiocarcinoma, which exhibits diffuse and invasive development. Types 3 and 4, which exhibit localized stenosis, should be carefully differentiated from cholangiocarcinoma using bile duct biopsy and intraductal ultrasonography (IDUS) 20. IgG4‐SC cases with stenosis at hilar hepatic lesions are sometimes associated with IgG4‐related inflammatory hepatic pseudotumors 15.

IgG4‐SC is also classified based on the presence or absence of association with AIP. IgG4‐SC is frequently associated with AIP. When IgG4‐SC is not associated with AIP, this is referred to as “isolated IgG4‐SC” 21. A Japanese multicenter study conducted by nine major hospitals of the IgG4‐related disease group of the Research Program of Intractable Diseases supported by the Ministry of Health, Labour, and Welfare of Japan reported that only 15 of 344 IgG4‐SC cases (4.4%) were not associated with AIP (2 of 246 type 1 IgG4‐SC cases [0.8%]; 5 of 56 type 2 IgG4‐SC cases [8.9%]; 8 of 42 type 3 and 4 IgG4‐SC cases [19.0%]) 22. Type 3 and 4 IgG4‐SC cases showed lower frequencies of association with AIP. A national survey performed in 2013 reported that isolated IgG4‐related SC included three cases of type 1, three cases of type 2, four cases of type 3, 22 cases of type 4, and 11 cases of other types 23. Type 4 is the most frequent type not associated with AIP. Isolated type 4 IgG4‐SC is very difficult to discriminate from cholangiocarcinoma. A case series reported five cases of isolated type 1 IgG4‐SC 24. Three of the five cases involved surgical resection of cholangiocarcinoma at the lower bile duct. The other two cases were diagnosed correctly as isolated type 1 IgG4‐SC, obviating the need for surgical resection based on high serum IgG4 levels and diffuse wall thickening in the bile duct.

Inclusion of the type 1 disease in the IgG4‐SC categories has been disputed. Some researchers claim that the type 1 disease should not be classified as IgG4‐SC for the following reasons:

1. The stricture of the lower bile duct is caused by compression due to AIP 25. This claim is based on the fact that type 1 IgG4‐SC was not found in some cases of focal‐type AIP with only body and tail involvement.
2. The frequency of IgG4‐SC increases when type 1 IgG4‐SC is included in the IgG4‐SC category in epidemiologic surveys.
3. In the international consensus diagnostic criteria, IgG4‐SC with extrapancreatic stenosis is regarded as additional organ involvement, which is useful in the diagnosis of AIP 26.

By contrast, others claim that type 1 IgG4‐SC should be included as a class of IgG4‐SC for the following reasons:

1. Pathologic examination of the bile duct wall obtained from surgically resected samples showed abundant infiltration of IgG4‐positive plasma cells, storiform fibrosis, and obstructive phlebitis, which are characteristics of IgG4‐SC inflammation 15.
2. Thickening of the bile duct wall is observed continuously from the intrapancreatic to extrapancreatic bile ducts 20.
3. Isolated type 1 IgG4‐SC exhibiting no AIP findings has been reported 24.

Indeed, it is difficult to identify the major factor contributing to the thickening of the bile duct wall – inflammation of the bile duct or compression due to AIP. Comments on diagnostic criteria of IgG4‐SC based on working group debates indicate that most cases of IgG4‐SC associated with AIP exhibit stricture of the lower bile duct caused by both thickening of the bile duct wall itself and pancreatic inflammation and edema 3.

#### What is the positioning of IgG4‐SC within SC?

- SC is classified as PSC, IgG4‐SC, or secondary SC according to the disease concept and clinical features. PSC is an idiopathic, progressive, and chronic intrahepatic cholestasis caused by fibrous stenosis of the intrahepatic and extrahepatic bile ducts and does not respond to steroid therapy, whereas IgG4‐SC does respond to steroid therapy. PSC is positioned different from secondary SC, which should be treated etiologically.

*Comment:* SC is classified as PSC, IgG4‐SC, or secondary SC according to the disease concept and clinical features. PSC is an idiopathic, progressive, chronic intrahepatic cholestasis caused by fibrous stenosis of the intrahepatic and extrahepatic bile ducts. To correctly diagnose PSC, IgG4‐SC and secondary SC must be ruled out 27, 28, 29. IgG4‐SC and PSC show similar imaging of the bile ducts, although their disease concept, pathophysiology, therapy, and prognosis are different; therefore, it is important to distinguish these two diseases.

IgG4‐SC has been reported in patients over age 60 years most commonly in association with an increase in serum IgG4 levels, whereas two peaks in age distribution were clearly observed in PSC patients with an approximately 10% increase in serum IgG4 levels 22, 23, 30. PSC is complicated by inflammatory bowel diseases such as ulcerative colitis. The prevalence of inflammatory bowel diseases in PSC patients is as high as 60–80% in Western countries and 34% in Japan 23, 31, 32. In contrast, IgG4‐SC is often complicated by AIP, sclerosing sialadenitis, and retroperitoneal fibrosis, but not inflammatory bowel diseases.

Pathologic characteristics of PSC include fibrous obliterative cholangitis, so‐called “onion‐skin lesions,” whereas IgG4‐positive plasma cells infiltrate the portal area in IgG4‐SC 33. IgG4‐SC responds to steroid therapy. In contrast, PSC is non‐responsive to steroids and immune‐suppressive agents, and currently, liver transplantation is the only effective treatment 34, 35, 36.

It is necessary to rule out the following features of secondary SC with obvious pathogenesis, including infection, cholangiocarcinomas, previous surgery or trauma involving the biliary tract, common bile duct stones and chronic inflammation, congenital biliary anatomic defects, corrosive cholangitis, ischemic bile duct stenosis, AIDS‐related cholangitis, and biliary injury caused by intra‐arterial chemotherapy 27, 31. Secondary SC should be principally treated etiologically 37, 38.

#### Are there characteristic histologic findings of IgG4‐SC?

- In intrahepatic and extrahepatic large bile ducts, transmural marked lymphoplasmacytic infiltration and fibrosis are observed, which give rise to bile duct wall thickening. Notably, storiform fibrosis and obliterative phlebitis are important histologic findings for diagnosing IgG4‐SC.
- An increase in IgG4‐positive cells is characteristic but not specific; thus, a diagnosis of IgG4‐SC needs to be established based on histologic findings.

**Figure 2:**
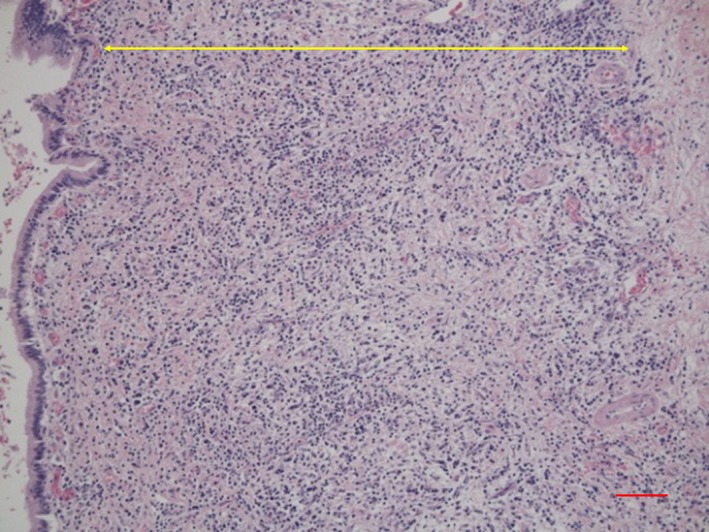
Histological findings of transmural inflammatory cell infiltration and fibrosis in the bile duct of IgG4‐SC (arrow). (Scale: 100 μm)

**Figure 3:**
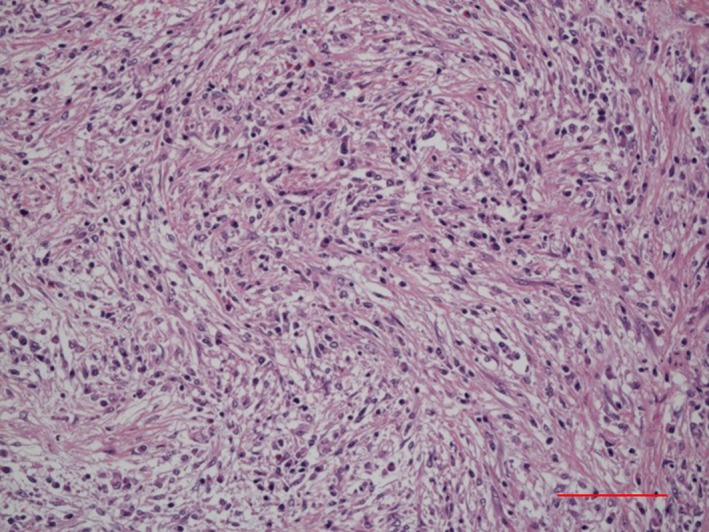
Histological findings of storiform fibrosis in the bile duct of IgG4‐SC. (Scale: 100 μm)

**Figure 4:**
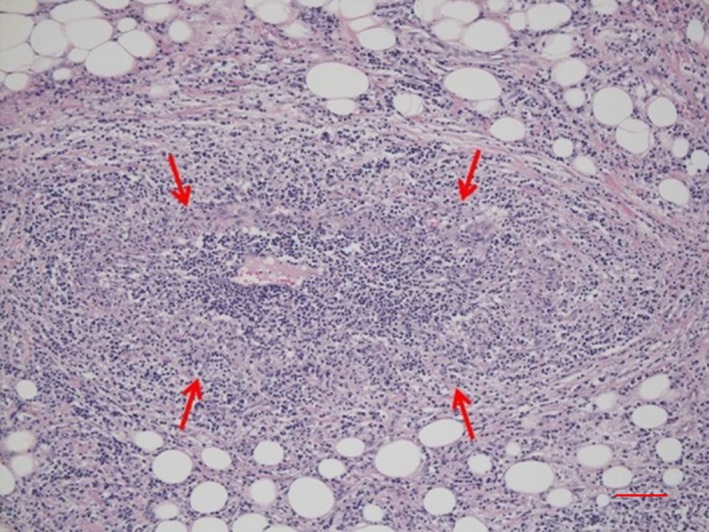
Histological findings of obliterative phlebitis (arrows) in the bile duct of IgG4‐SC. (Scale: 100 μm)

*Comment:* IgG4‐SC primarily involves the intrahepatic and extrahepatic large bile ducts and is characterized by transmural marked lymphoplasmacytic infiltration and fibrosis, which gives rise to duct wall thickening (Fig. 2) 15, 21, 39, 40, 41. In contrast to PSC, in which the focus of inflammation is the epithelium, no cell damage or inflammatory cell infiltration is observed in the epithelium. Similar to IgG4‐related disease in other organs, eosinophilic infiltration, storiform fibrosis (Fig. 3), and/or obliterative phlebitis (Fig. 4) are commonly identified, and the latter two are particularly regarded as diagnostically important. Storiform fibrosis is an irregular swirling arrangement of collagen 39, and inflammatory cells are commonly observed. Obliterative phlebitis is an inflammatory lesion with inflammatory cells and fibrosis that involves and obliterates the venous lumen 39. IgG4‐SC lesions involve not only the bile duct walls but also the peribiliary adipose tissue, peripheral nerves, and portal regions of the liver 15, 42. There are no histologic differences between IgG4‐SC with and without AIP 21, 42.

**Figure 5:**
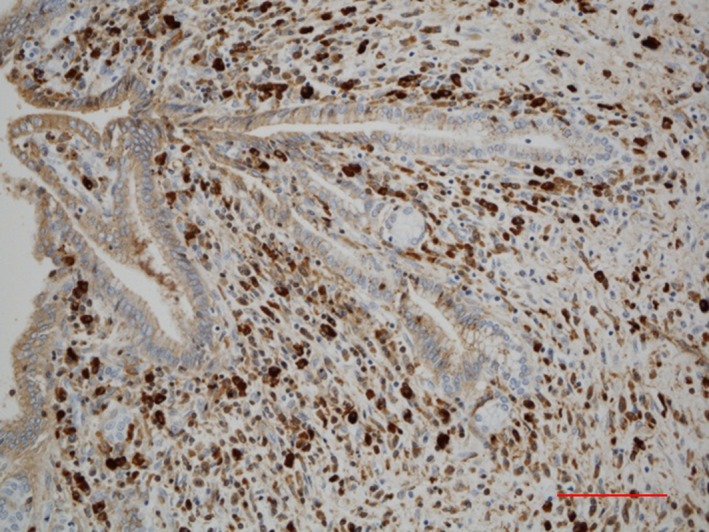
Immunohistochemical findings of infiltration of numerous IgG4‐positive cells in the bile duct of IgG4‐SC. IgG4‐immunostaining. (Scale: 100 μm)

Infiltration of numerous IgG4‐positive cells (Fig. 5) is characteristic of – but not specific to – IgG4‐SC. In PSC, numerous IgG4‐positive cells can also be identified, notably in the inflamed large bile ducts, with marked inflammatory cell infiltration 43, 44, 45. In a study of 122 explanted livers with PSC, >50 IgG4‐positive cells per high power field (HPF) were observed in 15.6% of the patients 45. In contrast to IgG4‐SC, however, IgG4‐positive cells are rare in the peripheral portal tracts in PSC. Numerous IgG4‐positive cells may also appear in cholangiocarcinomas 46, 47, and >50 IgG4‐positive cells per HPF were found in 9% of 54 resected specimens 46. Thus, the diagnosis of IgG4‐SC is difficult to establish based on IgG4‐immunostaining alone, and histologic findings must be thoroughly evaluated.

According to clinical diagnostic criteria of IgG4‐SC 2012 3, the diagnosis can be definitively made based on histologic findings if three of the following four conditions are identified: (1) marked lymphoplasmacytic infiltration and fibrosis; (2) >10 IgG4‐positive plasma cells per HPF; (3) storiform fibrosis; and (4) obliterative phlebitis. A cut‐off of >10 IgG4‐positive cells per HPF was adopted to make a biopsy‐based diagnosis of IgG4‐SC possible. In contrast, a consensus statement on the pathology of IgG4‐related disease 48 indicated that >50 IgG4‐positive cells per HPF in resected specimens and >10/HPF in biopsy samples as well as an IgG4/IgG‐positive cell ratio >40% need to be satisfied, and lesions are regarded as histologically highly suggestive of IgG4‐related disease if two of the following three conditions are additionally satisfied: (1) marked lymphoplasmacytic infiltration; (2) storiform fibrosis; and (3) obliterative phlebitis. IgG4‐SC cases that do not satisfy the requirement for an IgG4/IgG‐positive cell ratio >40% have been reported 21, however.

#### Is there any relationship between IgG4‐SC and hepatic inflammatory pseudotumors?

- IgG4‐SC sometimes presents with mass‐forming cholangitis, particularly around the hilar bile duct. These cases are referred to as hepatic inflammatory pseudotumors. The mass lesions consist of expanded periductal connective tissue with involvement of the liver parenchyma being uncommon.

*Comment:* Some patients with IgG4‐SC present with a hilar mass, imaging features of which mimic hilar cholangiocarcinomas 15, 49. Such cases are called IgG4‐related hepatic inflammatory pseudotumors. However, hepatic inflammatory pseudotumors are heterogeneous in nature and not always IgG4‐related. They are classified into lymphoplasmacytic and fibro‐inflammatory types based on histologic findings, with the former corresponding to IgG4‐related disease 50. Lymphoplasmacytic‐type pseudotumors typically develop in hilar bile ducts, whereas fibro‐inflammatory‐type lesions more commonly affect the liver parenchyma. Although a study based on surgically treated patients suggested that the lymphoplasmacytic type accounts for 37% of hepatic pseudotumors, the exact relative frequency is unknown 50.

#### What is the etiology of IgG4‐SC?

- Although the exact etiology is unknown, autoimmunity is the most suspected cause of IgG4‐SC.

*Comment*: Of several hypotheses that have been proposed as a potential pathogenetic mechanism of IgG4‐SC, an autoimmune theory is currently regarded as most likely correct. This possibility is supported by the occasional detection of autoantibodies in patients with IgG4‐SC, known disease‐susceptible HLA haplotypes for AIP, and good response to corticosteroids 9, 51. In addition, injection of IgG isolated from patients with AIP into neonatal mice was shown to induce pancreatic injury, suggesting a potential pathogenetic effect of IgG 52. An Italian research group raised the possibility that *Helicobacter pylori* infection initiates an immune reaction leading to the production of antibodies against the plasminogen‐binding protein of *Helicobacter pylori*, and these antibodies could behave as autoantibodies against the pancreatic acinar cells via molecular mimicry 53. However, another study could not validate these findings 54. Other potential pathogenetic models include allergy, paraneoplastic syndrome, and immune complex deposition 55, 56.

#### What is the epidemiology of IgG4‐SC?

- In Japan, the number of patients with IgG4‐SC is estimated at approximately 2,500, according to an epidemiologic study of AIP.

*Comment*: To date, no epidemiologic study of IgG4‐SC has been reported. Regarding AIP, another IgG4‐related disease, an epidemiologic study in 2011 estimated the prevalence and incidence as 4.6 and 1.4 per 100,000 population, respectively 57. As IgG4‐SC was present as a comorbidity in 39% of patients with AIP, the prevalence and incidence of IgG4‐SC in patients with AIP were extrapolated as 1.8 and 0.5 per 100,000 population, respectively. According to a nationwide survey for IgG4‐SC in 2015 19, approximately 10% of patients with IgG4‐SC are not diagnosed as having AIP. Collectively, the total prevalence of IgG4‐SC was estimated as 2.0 per 100,000 population, and the number of patients with IgG4‐SC was thus calculated as 2,500.

Case series of IgG4‐SC have been reported from the United States, the United Kingdom, and Japan (Table 1) 16, 19, 58. IgG4‐SC is a male‐dominant disease, with males accounting for 80% of all patients. All reports agree regarding the age at highest risk for developing IgG4‐SC, indicating an average or median age at diagnosis of 60–70 years. In case series from Japan, age at diagnosis ranged from 23.0 to 88.5 years, and no childhood or adolescent patients were reported 19, unlike in the case of PSC 23. Jaundice was the most prevalent symptom at diagnosis in reports from the United States and the United Kingdom, observed in more than 70% of all patients. Jaundice was the most prevalent symptom in Japan as well, but it was observed in only 35% of patients; notably, 28% of patients were diagnosed without any apparent symptoms 19. AIP was noted as a comorbidity in 90% of patients with IgG4‐SC in all case series.

**Table 1:** Comparison of clinical features of IgG4‐related sclerosing cholangitis

Region	Year	Number of cases	Male (%)	Age at diagnosis (years)^a^	Most prevalent symptom at diagnosis	Presence of AIP	Ref
United States	2008	53	85	62	Jaundice (77%)	92%	16
United Kingdom	2014	68	74	61	Jaundice (74%)	88%	58
Japan	2017	527	83	66	Jaundice (35%)	87%	19

*AIP* autoimmune pancreatitis

a Average (the United States), median (the United Kingdom, Japan)

#### Is the outcome of IgG4‐SC good?

- The outcome is probably excellent, as treatment with corticosteroids is effective in most cases of IgG4‐SC. However, as the long‐term outcome remains unclear, further studies are warranted.

*Comment:* The outcome of patients with IgG4‐SC has been investigated in retrospective cohorts in the United States 16, the United Kingdom 58, and Japan 19. Sample size, follow‐up period, corticosteroid treatment, and outcomes are summarized in Table 2.

**Table 2:** Comparison of treatment and prognosis of IgG4‐related sclerosing cholangitis

Region	Year	*n*	Follow‐up period (months)	Corticosteroid treatment	Cirrhosis	All‐cause mortality	LT	Mortality due to liver and bile duct diseases	Ref
United States	2008	53	29.5^a^	30 (57%)	4 (7.5%)	7 (13%)	0	1 (1.9%)	16
United Kingdom	2014	68	32.5	98 (85%)^b^	6 (5.2%)^a^	11 (9.6%)^a^	1	3 (2.6%)^a^	58
Japan	2017	527	49.2	458 (88%)	N/A	26 (5%)	0	4 (0.8%)	19

*LT* liver transplantation, *N/A* not available

a The average follow‐up period of patients treated with corticosteroids

b The proportion in 115 patients with autoimmune pancreatitis, including those without IgG4‐SC

Progression to cirrhosis was noted in 5.2% in the United Kingdom cohort and 7.5% in the United States cohort. Mortality due to liver or bile duct problems was found in only one case in the United States cohort and two cases (liver failure and cholangiocarcinoma) and one case involving liver transplantation in the United Kingdom. In Japan, only four cases (two cholangiocarcinoma and two hepatic failure) were recorded, accounting for 0.8%, which was lower than the United States and United Kingdom rates 19, 58. Therefore, poor outcomes, including liver or bile duct disease mortality and liver transplantation, can be avoided with corticosteroid treatment in most cases of IgG4‐SC, and the outcome is excellent. However, the follow‐up period remains insufficient to provide conclusive results, and potential biases associated with retrospective designs might be present in these case series. In this regard, the long‐term outcome remains unclear, and further studies are warranted.

In the United Kingdom report, the risk of all‐cause death was increased compared with matched national statistics (OR = 2.07, CI = 1.07–3.55, *P *=* *0.02). However, cancer‐related mortality was not increased compared with the national studies (incidence ratio 1.95, CI = 0.6–4.51, *P *=* *0.17); whether mortality is increased (and its cause if so) in patients with IgG4‐SC remains to be elucidated.

#### Is IgG4‐SC associated with the development of cholangiocarcinoma?

- IgG4‐SC is not considered a risk factor for cholangiocarcinoma because cholangiocarcinoma rarely occurs during the follow‐up period.

*Comment*: It is pathologically difficult to differentiate between cholangiocarcinoma associated with IgG4 and cholangiocarcinoma arising from IgG4‐SC, as 32% to 43% of patients with biliary tract cancer exhibit >10 IgG4‐positive plasma cells per HPF within and around cancerous nests 59. Three patients with cholangiocarcinoma and IgG4‐SC have been reported 60, 61, 62; two patients were diagnosed concurrently, and one patient was diagnosed with cholangiocarcinoma during the follow‐up period of IgG4‐SC treatment 62. Hirano et al. reported 14 patients with malignancy among 113 cases of IgG4‐related disease; however, cholangiocarcinoma occurred in only one patient 1.7 years after diagnosis of IgG4‐related disease (AIP) 63. Shiokawa et al. 55 also reported that among 108 patients with AIP, cholangiocarcinoma occurred in only one patient 5.6 years after the diagnosis during the follow‐up period; malignant disease occurred in 15 patients during the same period. A retrospective cohort study of 527 patients with IgG4‐SC in Japan 19 showed that four patients developed cholangiocarcinoma; two of the four patients were diagnosed at the same time, whereas the remaining two patients were diagnosed 4 months and 4 years after the diagnosis of IgG4‐SC, respectively.

Cholangiocarcinoma arising from IgG4‐SC is thought to be a rare complication. However, evaluating the risk of cholangiocarcinoma will require longer cohort studies, as IgG4‐SC is a relatively new concept for clinicians.

### Algorithm for diagnosis and treatment of IgG4‐SC (Figs 6, 7, 8, 9)

**Figure 6:**
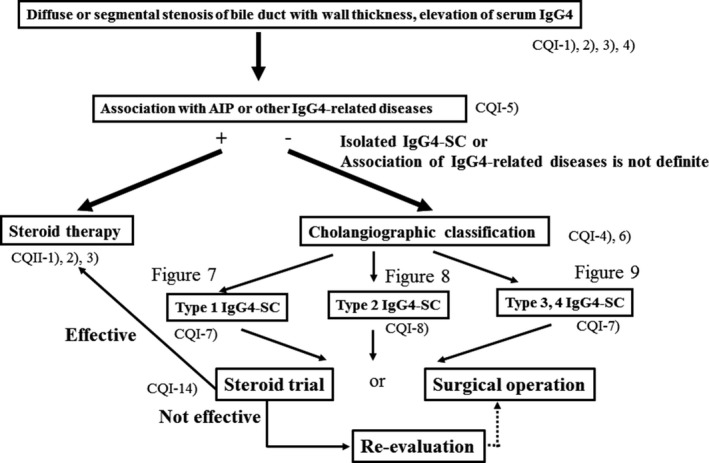
Algorithm for diagnosis and treatment of IgG4‐SC referred from Reference 18

**Figure 7:**
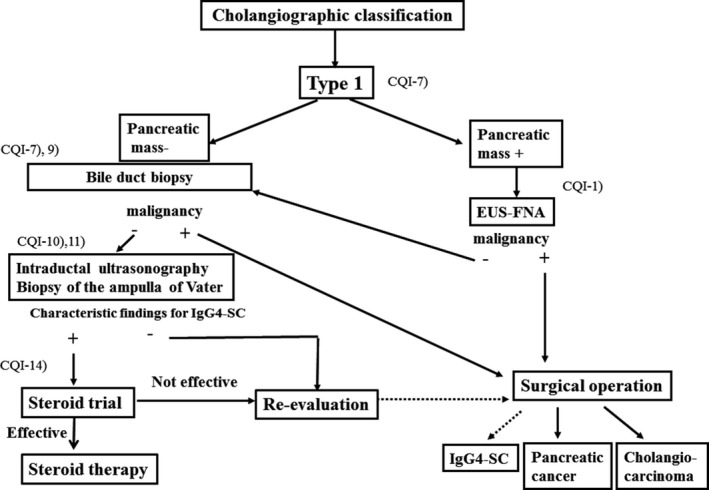
Algorithm for diagnosis and treatment of IgG4‐SC referred from Reference 18

**Figure 8:**
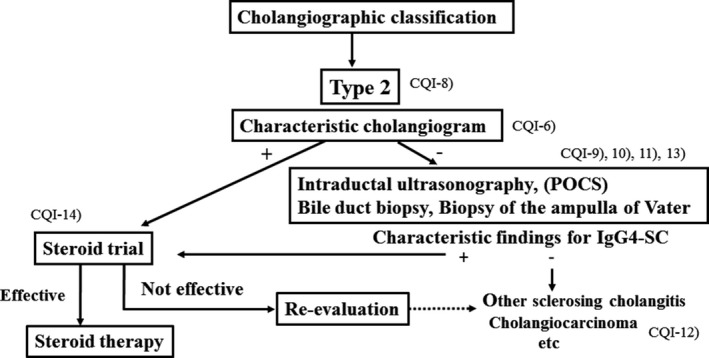
Algorithm for diagnosis and treatment of IgG4‐SC referred from Reference 18

**Figure 9:**
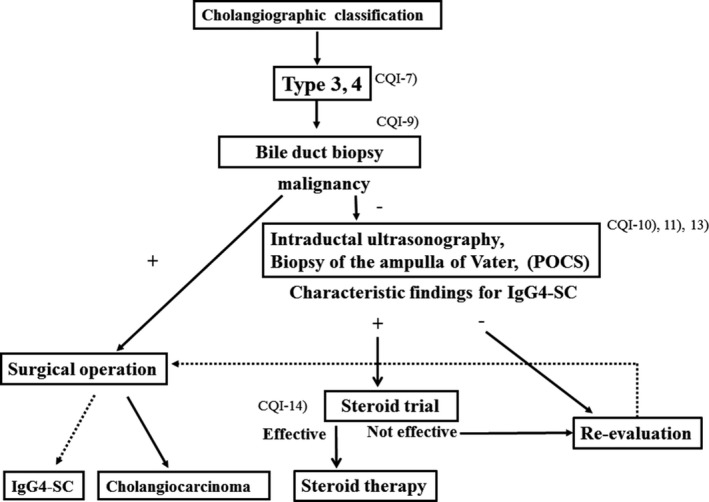
Algorithm for diagnosis and treatment of IgG4‐SC referred from Reference 18

IgG4‐SC is suspected when diffuse or segmental stenosis of the bile duct with wall thickening is seen and/or the serum IgG4 level is high. The diagnosis of IgG4‐SC is based on clinical diagnostic criteria of IgG4‐SC 2012. When associated with AIP or other IgG4‐related diseases, the diagnosis is straightforward. If not or association of these diseases is not definite, differential diagnosis can be difficult due to the challenge of obtaining a sufficient sample for the diagnosis of IgG4‐SC by bile duct biopsy. IgG4‐SC cholangiograms are similar to those for PSC and cholangiocarcinoma. Type 1 IgG4‐SC produces stenosis only in the lower bile duct (intrapancreatic duct) and thus should be differentiated from pancreatic cancer and cholangiocarcinoma. Type 2 IgG4‐SC, in which stenosis is diffusely distributed throughout the intrahepatic and extrahepatic bile ducts, should be differentiated from PSC. Type 3 IgG4‐SC is characterized by stenosis in the hilar hepatic lesions and lower bile duct. Type 4 IgG4‐SC presents with strictures of the bile duct only in the hilar hepatic lesions. The cholangiographic findings of types 3 and 4 IgG4‐SC should be discriminated from those of cholangiocarcinoma (Fig. 6).

In the diagnosis of type 1 IgG4‐SC, pancreatic cancer should be ruled out by endoscopic ultrasonography‐guided fine‐needle aspiration (EUS‐FNA) when the pancreatic mass of pancreas head is detected. Then, cholangiocarcinoma should be ruled out by bile duct biopsy under ERC. When IDUS and/or biopsy of the ampulla of Vater shows characteristic findings for IgG4‐SC, steroid trial is suggested. If these examinations do not show characteristic findings or steroid trial is not effective, re‐evaluation or surgical operation is selected. Surgical operation is indicated when malignant cells are detected or malignant diseases cannot be ruled out after careful re‐evaluation (Fig. 7).

In the diagnosis of type 2 IgG4‐SC, when characteristic cholangiogram is obtained, steroid trial is suggested. If combination of IDUS, peroral cholangioscopy (POCS), bile duct biopsy and biopsy of the ampulla of Vater reveals characteristic findings for IgG4‐SC in cases without characteristic cholangiogram, steroid trial is also suggested. If these examinations do not show characteristic findings or steroid trial is not effective, other sclerosing cholangitis or cholangiocarcinoma should be considered (Fig. 8).

In the diagnosis of types 3 and 4 IgG4‐SC, cholangiocarcinoma should be ruled out by bile duct biopsy under ERC. If malignant cells are not detected and IDUS, POCS and/or biopsy of the ampullary of Vater shows characteristic findings for IgG4‐SC, steroid trial is suggested. If these examinations do not show characteristic findings or steroid trial is not effective, re‐evaluation or surgical operation is selected. Surgical operation is indicated when malignant cells are detected or malignant diseases cannot be ruled out after careful re‐evaluation (Fig. 9).

### Summary of imaging findings suggestive of IgG4‐SC (Fig. 10)

**Figure 10:**
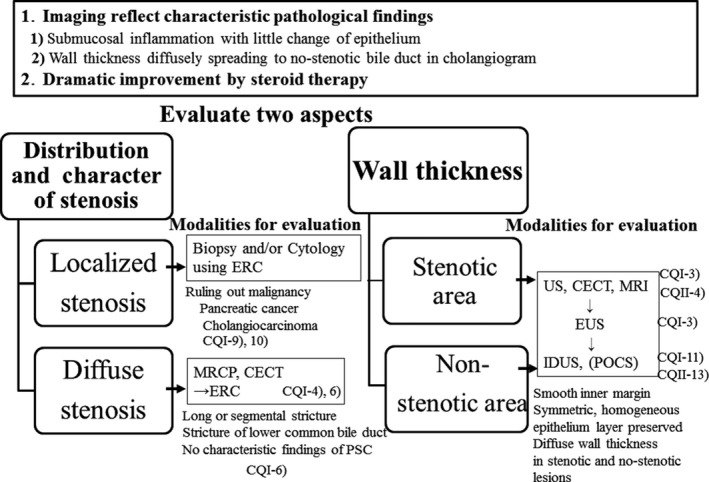
Summary of imaging findings suggestive of IgG4‐SC

Imaging reflects the characteristic pathologic findings, such as submucosal inflammation with little change in the epithelium and wall thickening diffusely spreading to non‐stenotic bile ducts in cholangiograms. In addition, these findings are dramatically improved with steroid therapy.

Two aspects should be evaluated: distribution and character of stenosis, and wall thickness.

#### Distribution and character of stenosis

Ruling out malignant diseases using biopsy and cytology under ERC is necessary in localized stenosis.

Evaluation of cholangiographic differences is necessary in diffuse stenosis.

#### Wall thickness

Diffuse wall thickening with preserved epithelium in stenotic and non‐stenotic lesions is a characteristic finding for IgG4‐SC.

### Treatment of IgG4‐SC (Fig. 11)

**Figure 11:**
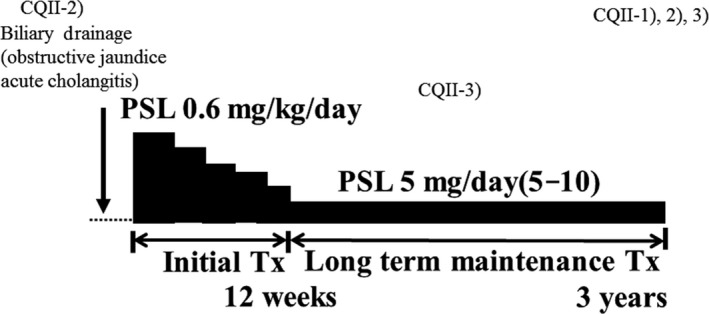
Schematic illustration of treatment of IgG4‐SC

Oral prednisolone at 0.6 mg/kg/day is recommended for initial remission induction therapy, with gradual reduction to a maintenance dose of 5 mg/day by 2–3 months. Maintenance dose should be continued for at least 3 years.

Patients with comfortable radiological and serological improvement can stop steroids. After treatment is discontinued, patients should be followed up for relapse.

## Results

### Clinical questions

#### Diagnosis

##### CQI‐1) How is IgG4‐SC diagnosed?

- We recommend that diagnosis of IgG4‐SC be based on the following four criteria: characteristic biliary imaging findings, elevation of serum IgG4 levels, coexistence of IgG4‐related diseases, except those of the biliary tract, and characteristic histopathologic features (Recommendation 1, level C).
- In a specialized facility in which detailed examinations such as endoscopic biliary biopsy and EUS‐FNA can be performed, we suggest evaluating the effectiveness of steroid therapy as an optional extra diagnostic criterion to confirm a diagnosis of IgG4‐SC after negative work‐up for malignancy (Recommendation 2, level D).

*Comment*: As IgG4‐SC exhibits various cholangiographic features similar to those of PSC, pancreatic cancer, and cholangiocarcinoma, the differential diagnosis of IgG4‐SC from these three progressive or malignant diseases is very important.

Two sets of diagnostic criteria for IgG4‐SC have been proposed. The HISORt (histology, imaging, serology, other organ involvement, and response to steroid therapy) criteria were originally developed for AIP and adapted for IgG4‐SC 16. In 2012, a second set of diagnostic criteria for IgG4‐SC was proposed by a Japanese group 3 (Table 3). Diagnosis of IgG4‐SC is based on the following four criteria: (1) characteristic biliary imaging findings; (2) elevation of serum IgG4 levels; (3) coexistence of IgG4‐related diseases, except those of the biliary tract; and (4) characteristic histopathologic features. As it is sometimes difficult to obtain sufficient biopsy specimens of the bile duct from patients suffering from IgG4‐SC, evaluation of the effectiveness of steroid therapy is an optional extra diagnostic criterion to confirm a diagnosis of IgG4‐SC. However, efforts should be made to collect sufficient tissue samples for diagnosis, and easy steroid trials should be avoided. The effectiveness of steroid therapy should be cautiously evaluated, as some malignant lesions improve after steroid administration 64. If neoplastic lesions cannot be clinically ruled out after steroid therapy, a re‐evaluation should be performed to rule out malignant cholangiopancreatic diseases.

**Table 3:** Clinical diagnostic criteria of IgG4‐related sclerosing cholangitis 2012 3

**1. Diagnostic items**	
(1) Biliary tract imaging reveals diffuse or segmental narrowing of the intrahepatic and/or extrahepatic bile duct associated with the thickening of bile duct wall.	
(2) Hematological examination shows elevated serum IgG4 concentrations (≥135 mg/dl).	
(3) Coexistence of autoimmune pancreatitis, IgG4‐related dacryoadenitis/sialadenitis, or IgG4‐related retroperitoneal fibrosis.	
(4) Histopathological examination shows:	
a. Marked lymphocytic and plasmacyte infiltration and fibrosis	
b. Infiltration of IgG4‐positive plasma cells: >10 IgG4‐positive plasma cells/HPF	
c. Storiform fibrosis	
d. Obliterative phlebitis	
Option: Effectiveness of steroid therapy	
A specialized facility, in which detailed examinations such as endoscopic biliary biopsy and endoscopic ultrasound‐guided fine needle aspiration (EUS‐FNA) can be administrated, may include in its diagnosis the effectiveness of steroid therapy, once pancreatic or biliary cancers have been ruled out.	
**2. Diagnosis**	
Definite diagnosis:	(1) + (3)
	(1) + (2) + (4) a, b
	(4) a, b, c
	(4) a, b, d
Probable diagnosis:	(1) + (2) + Option
Possible diagnosis:	(1) + (2)

It is necessary to exclude PSC, malignant diseases such as pancreatic or biliary cancers, and secondary sclerosing cholangitis caused by the diseases with obvious pathogenesis. If IgG4‐SC cannot be clinically ruled out, a patient must not be treated with facile steroid therapy but should be referred to a specialized medical facility.

IgG4‐SC is frequently associated with AIP 16, and this association is useful for diagnosis of IgG4‐SC 1. However, it is particularly difficult to accurately diagnose IgG4‐SC in the absence of AIP 21, 24, 65. Occasionally, IgG4‐SC is associated with other systemic IgG4‐related diseases, including IgG4‐related dacryoadenitis/sialadenitis, IgG4‐related retroperitoneal fibrosis, and IgG4‐related kidney disease 66. These associations can also assist diagnosis of IgG4‐SC. We use diagnostic criteria for IgG4‐SC for diagnosis of IgG4‐SC 3, and we use comprehensive diagnostic criteria for IgG4‐related disease 2011 67 for diagnosis of other IgG4‐related diseases without organ specific diagnostic criteria. In contrast to PSC, inflammatory bowel disease is rarely observed in patients with IgG4‐SC 68.

It should be noted that an elevated serum IgG4 level is also observed in conditions such as atopic dermatitis, pemphigus, and asthma; in particular, elevated serum IgG4 levels are observed in PSC and some malignant cholangiopancreatic diseases (e.g. pancreatic cancer and cholangiocarcinoma) 22, 69, 70.

IgG4‐SC with strictures of the bile duct in the hilar hepatic lesions must be discriminated from hilar cholangiocarcinoma. In these cases, endoscopic procedures such as endoscopic ultrasonography (EUS), IDUS, cytologic examination, and/or biopsy of the bile duct are useful for correct diagnosis 21, 24, 65.

##### CQI‐2) Is an examination of serum IgG4 level recommended for diagnosing IgG4‐SC?

- We recommend an examination of serum IgG4 levels for the diagnosis of IgG4‐SC (Recommendation 1, level C).
- We suggest an examination of serum IgG4 levels for differential diagnosis from cholangiocarcinoma (Recommendation 2, level D).
- We suggest an examination of serum IgG4 levels for differential diagnosis from PSC (Recommendation 2, level D).

*Comment*: An elevation of serum IgG4 levels is seen in 90% of IgG4‐SC cases 22, 71. Conversely, no elevation of serum IgG4 level is seen in 10% of cases 22, 71. However, as an elevation of serum IgG4 levels is also seen in 8–14% of cholangiocarcinoma cases 22, 69, IgG4‐SC cannot be diagnosed based solely on an elevated serum IgG4 level (sensitivity 64–90%, specificity 87–93%) 22, 69. A cut‐off of ≥206 mg/dL could be useful for types 3 and 4 IgG4‐SC to distinguish them from cholangiocarcinoma 22 because types 3 and 4 IgG4‐SC are known to mimic cholangiocarcinoma.

An elevation of serum IgG4 levels is also seen in 9–22% of PSC cases 22, 23, 43, 70, 71, 72, 73, 74, 75, 76, 77. It was reported that a cut‐off of ≥117 mg/dl has a sensitivity of 92% and specificity of 88% 22; with a cut‐off of ≥140 mg/dl, it had a sensitivity of 90% and specificity of 85%; and with a cut‐off of ≥280 mg/dl, it had a sensitivity of 70% and specificity of 98% 71. The optimal cut‐off value was reported to be 250 mg/dl, with a sensitivity of 67–89% and specificity of 95% 71.

Serum IgG4 has been assayed by two world‐wide systems, Binding‐Site system 22 and Siemens system 71. In Japan, only Binding‐Site system is available, whereas in Europe and the United States, both Binding‐Site and Siemens systems are used. Serum IgG4 levels of Siemens system was reported to be 60% higher than those of Binding‐Site system due to difference of standards for both systems 72. Accordingly, serum IgG4 assay with Siemens system may bring about false positive, if cut‐off of Binding‐Site system (≥140 mg/dl), is applied 71.

An elevation of serum IgG4 levels is seen in most cases of IgG4‐SC, so it is useful for the diagnosis of IgG4‐SC. However, an elevation of serum IgG4 levels is not specific for the diagnosis of IgG4‐SC. Cholangiocarcinoma and PSC cannot be excluded solely based on an elevation of serum IgG4 levels.

##### CQI‐3) Is ultrasonography (including EUS) recommended for diagnosing IgG4‐SC?

- We suggest ultrasonography for diagnosis of IgG4‐SC (Recommendation 2, level D).
- We suggest EUS for differential diagnosis from cholangiocarcinoma (Recommendation 2, level D).

**Figure 12:**
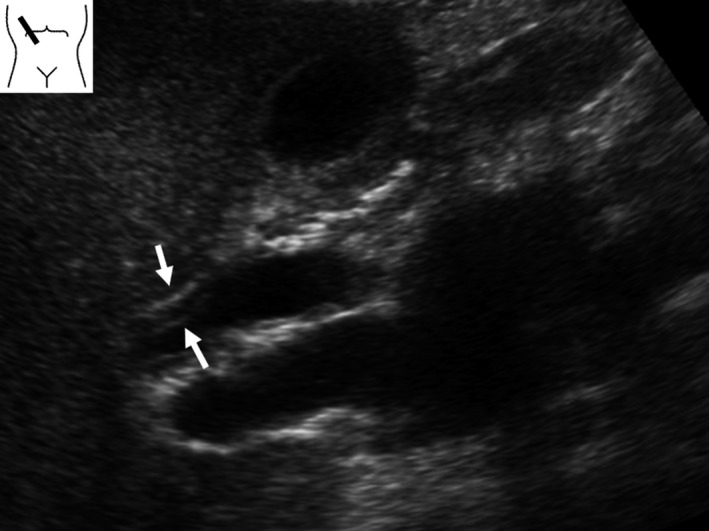
Ultrasonographic image of IgG4‐SC. Ultrasonography detects marked wall thickening in the extrahepatic bile duct (arrows)

*Comment*: Only a few reports have described ultrasonographic findings for IgG4‐SC 78, 79, 80, 81. Before the disease concept of IgG4‐SC was established, Kamisawa et al. reported observing bile duct and gallbladder wall thickening in 60% of patients with AIP 78. Koyama et al. reported characteristic bile duct and gallbladder wall thickening (Fig. 12) on ultrasonography in 37.8% of patients with AIP 79. Characteristic wall thickening was reported as laminar structures or low echoic thickening of the wall 80, 81. Koyama et al. 79 divided patients into two groups according to ultrasonographic findings of bile duct wall thickening: (1) three‐layer type, involving marked wall thickening apparent on ultrasonography as high‐low‐high echo of the bile duct wall; and (2) parenchymal‐echo type, involving a thickened wall that occupies the entire lumen of the bile duct with appearance of parenchymal echo in the bile duct. They also reported rapid improvement in bile duct stenosis after steroid therapy but delayed improvement of the bile duct and gallbladder wall thickening 79.

**Figure 13:**
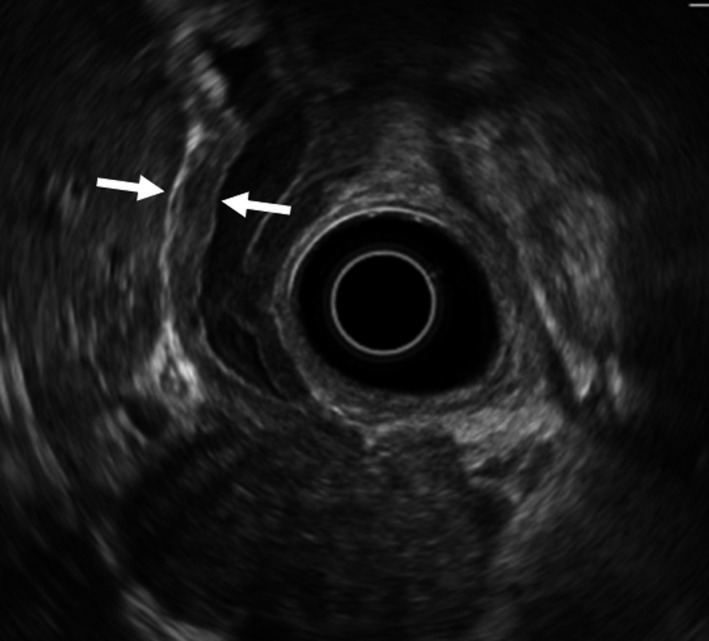
EUS image of IgG4‐SC. EUS shows a homogeneous, high‐low‐high echo of the bile duct wall thickening (arrows)

Ultrasonography can detect wall thickening from the intrahepatic to extrahepatic bile ducts but cannot detect wall thickening of the intrapancreatic bile duct. It is difficult to distinguish IgG4‐SC from PSC or cholangiocarcinoma using only ultrasonography. Nakamura et al. reported that the characteristic ultrasonographic findings of PSC include a homogeneous, high‐low‐high echo of the bile duct wall thickening, and in cases of severe wall thickening, the lumen of the bile duct becomes indistinct 82. These findings are similar to those of IgG4‐SC (Fig. 13); thus, the differential diagnosis of both is difficult. Du et al. reported ultrasonographically determined wall thickening in 3 of 21 (14.3%) patients with IgG4‐SC, occupying lesions in seven patients (33%), and dilation in 18 patients (85.7%). In contrast, ultrasonography revealed wall thickening in 11 of 194 (5.7%) patients with cholangiocarcinoma, occupying lesions in 70 patients (36.1%), and dilation in 137 patients (70.6%). There were no significant differences between IgG4‐SC and cholangiocarcinoma 83. In one case report, ultrasonography detected a low echoic tumor in the liver, and the patient was diagnosed as having IgG4‐SC based on pathologic examinations of resected specimens 84. It is difficult to diagnose IgG4‐SC using only ultrasonography.

Ultrasonography is a useful modality for detecting wall thickening of the bile duct in patients with IgG4‐SC. Furthermore, ultrasonography can detect pancreatic swelling at the same time in patients with AIP. As ultrasonography is non‐invasive, it is proposed for use in the diagnosis of IgG4‐SC.

Few reports have described EUS findings of IgG4‐SC 83, 85, 86. Du et al. 83 reported the EUS revealed wall thickening in 17 of 18 (94.4%) patients with IgG4‐SC, occupying lesions in one patient (5.6%), and dilation in 10 patients (55.6%). On the other hand, EUS revealed wall thickening in only 3 of 10 (30%) patients with cholangiocarcinoma, occupying lesions in eight patients (80%), and dilation in five patients (50%). There were significant differences between IgG4‐SC and cholangiocarcinoma 83. Du et al. reported observing thickened walls significantly more frequently in IgG4‐SC cases than in cholangiocarcinoma, and significantly more occupying lesions were observed in cholangiocarcinoma than IgG4‐SC cases 83. They concluded that EUS is an effective imaging tool for diagnosis of IgG4‐SC and cholangiocarcinoma 83.

Naitoh et al. reported that thickened walls detected by ultrasonography, computed tomography (CT), EUS, and IDUS involve circular‐symmetrical thickening, smooth outer and inner margins, and homogeneous internal echo at the stenotic area of the bile duct 86. Sometimes, wall thickening is observed in the bile duct without obvious stenosis and wall thickening is observed in the gallbladder, too. Because EUS can detect characteristic findings of IgG4‐SC and thus facilitate a differential diagnosis from cholangiocarcinoma, EUS is proposed for use in differential diagnosis from cholangiocarcinoma.

##### CQI‐4) Are CT and magnetic resonance imaging (MRI) recommended for diagnosing IgG4‐SC?

- In order to detect dilation or stenosis of bile ducts and obtain a complete image of the biliary system, we suggest contrast‐enhanced CT and MRI/magnetic resonance cholangiopancreatography (MRCP) (Recommendation 2, level D).
- For differential diagnosis of PSC or cholangiocarcinoma, we suggest contrast‐enhanced CT and MRI/MRCP (Recommendation 2, level D).

**Figure 14:**
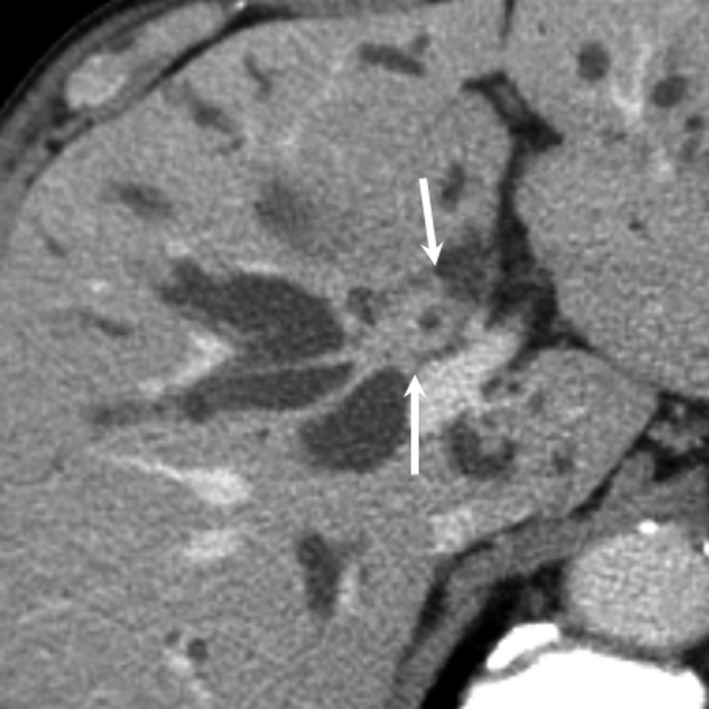
CT image of IgG4‐SC. Contrast‐enhanced CT (delayed image) demonstrates concentric wall thickening of the hilar bile duct (arrows)

**Figure 15:**
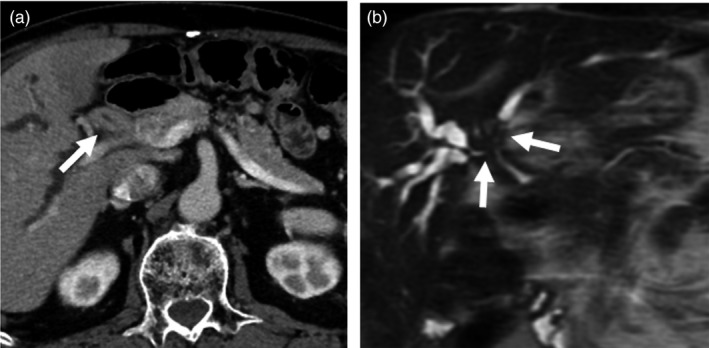
CT and MRCP images of IgG4‐SC. (**a**) In CT, the hilar bile duct shows marked thickening (arrow). (**b**) MRCP demonstrates bile duct strictures in the hilar bile duct (arrows)

*Comment*: CT and MRI enable objective detection of biliary abnormalities, including dilatation or wall thickening of bile ducts, and are considered useful for identifying bile duct lesions (Fig. 14). Additionally, T2‐weighted images or MRCP enable non‐invasive imaging of the complete biliary system and determination of the distribution or degree of biliary strictures. As such, CT and MRI are considered useful for providing clues that can aid in the diagnosis of IgG4‐SC (Fig. 15). In terms of differential diagnosis of other biliary diseases, such as PSC or cholangiocarcinoma in particular, CT and MRI have both advantages and limitations. In IgG4‐SC, biliary inflammation occurs transmurally, preserving the epithelial layer almost intact. In contrast, PSC mainly involves the luminal side, including the epithelium, and cholangiocarcinoma arises from the epithelial layer and grows invasively 39, 87. There are several differences regarding the primary locations of these conditions; however, it is challenging to demonstrate these differences by CT or MRI. To date, characteristic reported imaging findings for IgG4‐SC include: (1) concentric bile duct wall thickening extending along the long axis; (2) smooth inner/outer margins; (3) visibility of patent bile duct in the strictures and mildness of upper bile duct dilatation; and (4) continuous bile duct wall thickening covering intrahepatic and extrahepatic bile ducts. However, the prevalence of these findings varies depending on the report; more reliable findings other than the abnormalities in the pancreas (presence of AIP) have yet to be reported 87, 88, 89, 90, 91. Although Kim et al. reported that MRCP can detect characteristic findings for PSC, such as multiple intrahepatic bile duct stenosis or pruned‐tree appearance, the spatial resolution of MRCP falls short of that of ERC 92. Furthermore, for detailed evaluation of bile duct wall thickening, EUS or IDUS provides more information than MRCP. However, considering the ability to non‐invasively obtain a complete image of the biliary and pancreatic ducts and evaluate the concentricity of bile duct wall thickening, the degree of upstream bile duct dilatation, and the invasiveness of the lesion, it is reasonable to consider that contrast‐enhanced CT or MRI/MRCP could contribute to the diagnosis of IgG4‐SC when compared with ERC or IDUS. Additionally, IgG4‐SC is usually accompanied by extra‐bile duct lesions, the presence of which can sometimes contribute to the diagnosis 93. For systemic screening of IgG4‐related lesions, it is worth performing contrast‐enhanced CT. Furthermore, adding high‐contrast‐resolution MRI (fat‐suppressed T1‐weighted, or diffusion‐weighted images) or MRCP, which can provide complete imaging of the pancreas ducts, can sometimes reveal pancreatic lesions (e.g. AIP) not apparent in contrast‐enhanced CT scans. CT and MRI/MRCP are relatively well‐known diagnostic modality for general population. CT and MRI scanners are distributing nationwide and available for all patients. Although imaging protocol and quality for biliary imaging have been standardized, radiation exposure should be concerned in repeated CT examinations. Consequently, when IgG4‐SC is suspected in clinical settings, systemic screening using contrast‐enhanced CT and detailed evaluation of the bile ducts and pancreas using MRI/MRCP could aid in the diagnosis.

##### CQI‐5) Is consideration of other associated diseases useful for diagnosing IgG4‐SC?

- AIP is the disease most frequently associated with IgG4‐SC, recognized in about 90% of patients with IgG4‐SC. Apart from AIP, IgG4‐SC is associated with dacryoadenitis, sialadenitis, retroperitoneal fibrosis, kidney lesions, pulmonary lesions, lymph node lesions, and vascular lesions (aorta and coronary arteries). We suggest consideration of these IgG4‐related lesions (Recommendation 2, level D).

**Figure 16:**
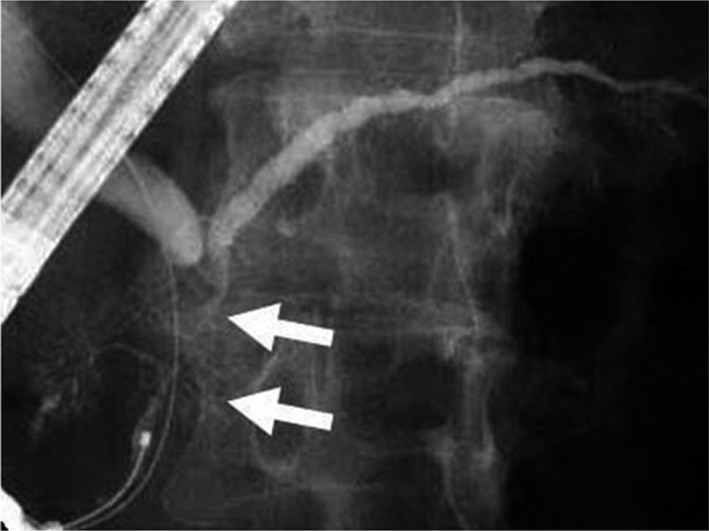
ERCP image of a patient with AIP in which the lesion was limited to the pancreatic head. Narrowing of the main pancreatic duct in the head (arrows) and strictures of the lower bile ducts are recognized

**Figure 17:**
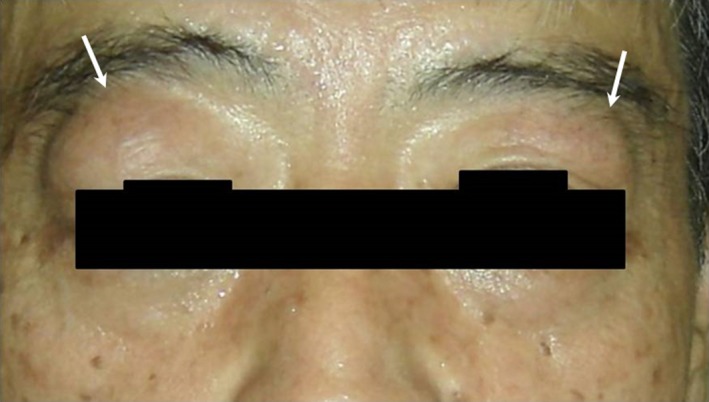
IgG4‐related dacryoadenitis. Swelling of right and left lacrimal glands (right > left) is recognized (arrows)

**Figure 18:**
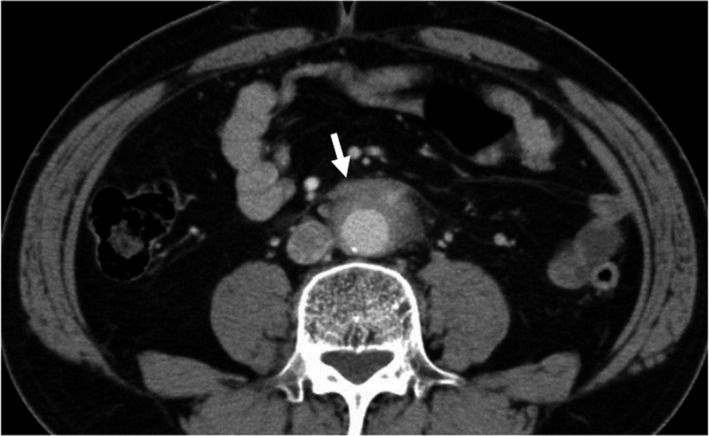
CT image of IgG4‐related retroperitoneal fibrosis. Soft‐tissue mass surrounding the abdominal aorta is recognized (arrow)

*Comment:* Although there are not many reports describing analyses of a large number of patients with IgG4‐SC, the most frequent associated disease is AIP (87–92%) 16, 19. On the other hand, IgG4‐SC is also the disease most frequently associated with AIP. Lower bile duct strictures are observed in about 80% of patients with AIP. In AIP cases with pancreatic head lesions, lower bile duct strictures often occur because of pancreatitis or pancreatic edema; therefore, the association between IgG4‐SC and AIP may be very close (Fig. 16) 25, 94. In addition to AIP, IgG4‐SC is known to be associated with dacryoadenitis (Fig. 17), sialadenitis, retroperitoneal fibrosis (Fig. 18), as well as kidney, pulmonary, lymph node, and vascular (aorta and coronary artery) lesions. However, an association with inflammatory bowel diseases is rare 16, 19, 23. Considering that IgG4‐SC is a systemic IgG4‐related disease, all lesions regarded as IgG4‐related disease may be associated diseases of IgG4‐SC. That is, associated diseases of IgG4‐SC can occur in various organs, including the central nervous system (hypophysitis and hypertrophic pachymeningitis), thyroid, liver, gastrointestinal tract, prostate, eyes, skin, and mammary glands 67.

##### CQI‐6) Is ERC recommended for diagnosing IgG4‐SC?

- ERC shows diffuse or segmental stricture of the intrahepatic and/or extrahepatic bile ducts (Recommendation 2, level D).
- ERC is useful for differentiation between IgG4‐SC and PSC (Recommendation 2, level D).

*Comment*: Biliary tract imaging in IgG4‐SC reveals diffuse or segmental stricture of the intrahepatic and/or extrahepatic bile ducts. Although MRCP provides useful information, stricture of the bile duct should be assessed by direct cholangiography (i.e. by ERC or percutaneous transhepatic cholangiography). In clinical practice, ERC is recommended as the most useful diagnostic imaging modality for diagnosing IgG4‐SC 3, 95.

**Figure 19:**
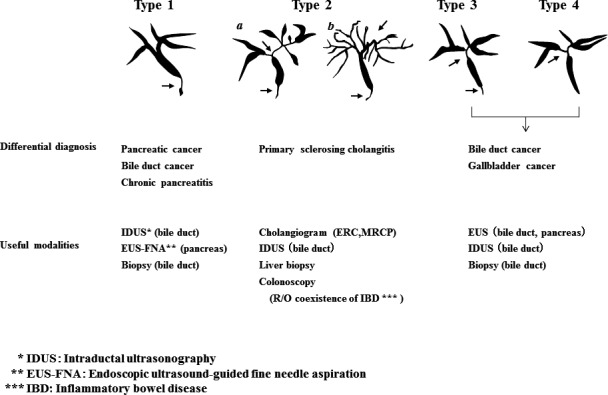
Cholangiographic classification of IgG4‐SC and the differential diagnosis of IgG4‐SC referred from Reference 3

The characteristic features of IgG4‐SC can be classified into four types based on the region of strictures as revealed by cholangiography and differential diagnosis (Fig. 19) (refer to Fig. 1).

**Figure 20:**
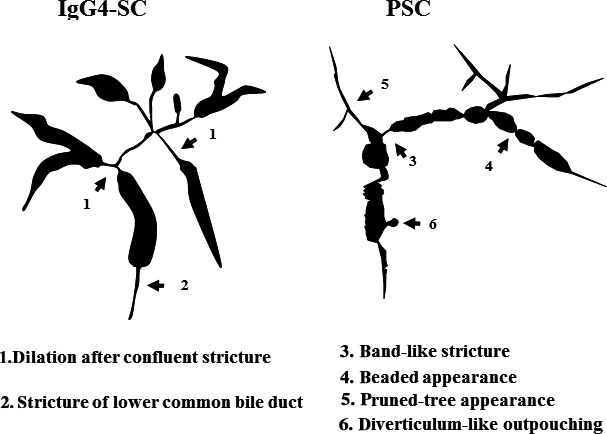
Schematic illustration comparing the cholangiographic findings in PSC and IgG4‐SC referred from Reference 3

**Figure 21:**
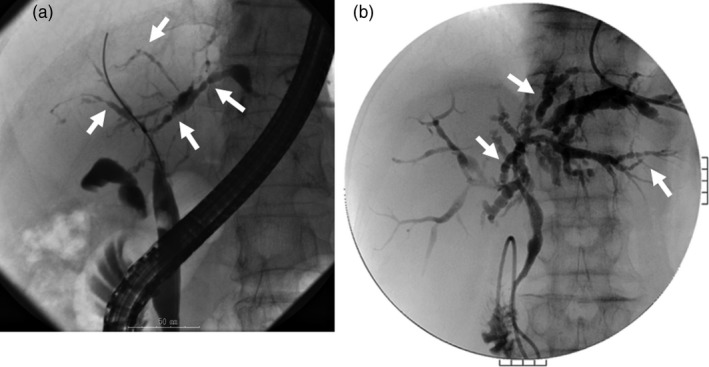
Cholangiogram of PSC showing (**a**) band‐like strictures and (**b**) beaded appearance (arrows)

**Figure 22:**
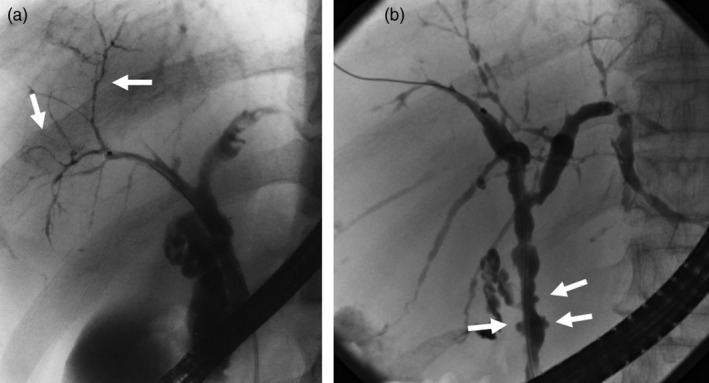
Cholangiogram of PSC showing (**a**) pruned‐tree appearance and (**b**) diverticulum‐like outpouching (arrows)

**Figure 23:**
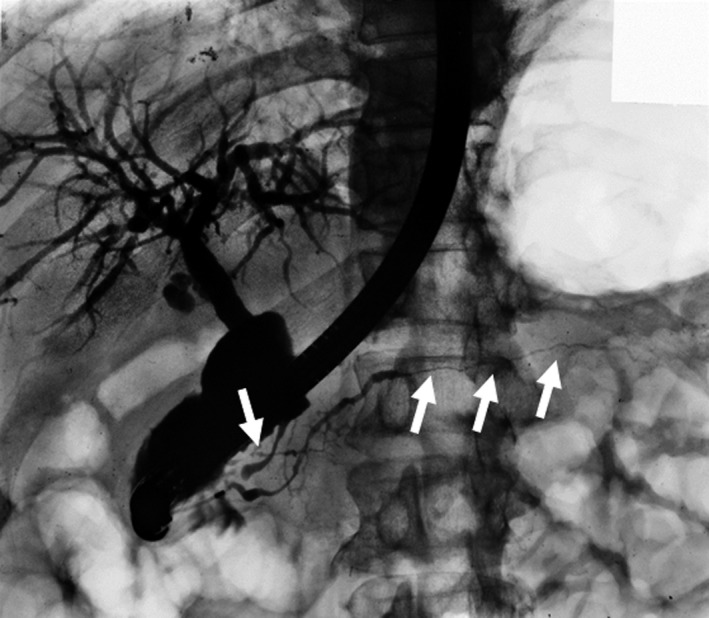
Cholangiopancreatogram of type 1 IgG4‐SC showing the lower bile duct stricture and irregular narrowing of the main pancreatic duct (arrows)

**Figure 24:**
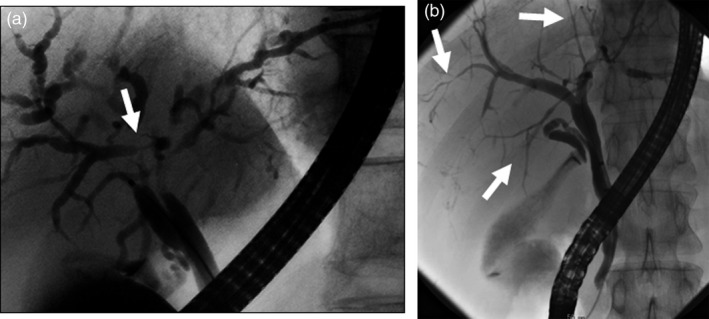
Cholangiogram of (**a**) type 2a IgG4‐SC showing dilatation after a confluent stricture (arrow) and (**b**) type 2b IgG4‐SC showing no dilatation after a confluent stricture (arrows)

The main cause of biliary strictures in IgG4‐SC is severe lymphoplasmacytic infiltration into the bile ducts in the long region, resulting in long strictures. The main cause of the biliary stricture in PSC, by comparison, is obliterative fibrosis, which results in short strictures and bile duct loss 27, 95, 96 (Fig. 20). The characteristic cholangiographic findings of IgG4‐SC may allow differentiation between IgG4‐SC and PSC. The dominant cholangiographic findings of PSC, including band‐like strictures (short strictures of 1–2 mm) (Figs 20, 21a), beaded appearance (Figs 20, 21b), pruned‐tree appearance (Figs 20, 22a), or diverticulum‐like outpouching (Figs 20, 22b), are rarely observed in IgG4‐SC. By contrast, the characteristic cholangiographic findings of IgG4‐SC are lower bile duct stricture (Figs 20, 23) and dilatation after a confluent stricture (Figs 20, 24) 96, 97. Sometimes it is difficult to distinguish between type 2b IgG4‐SC and PSC based on the cholangiographic findings when the only cholangiographic finding in PSC is a pruned‐tree appearance. When IgG4‐SC is associated with AIP, pancreatography shows narrowing of the main pancreatic duct, which is characteristic of AIP.

**Figure 25:**
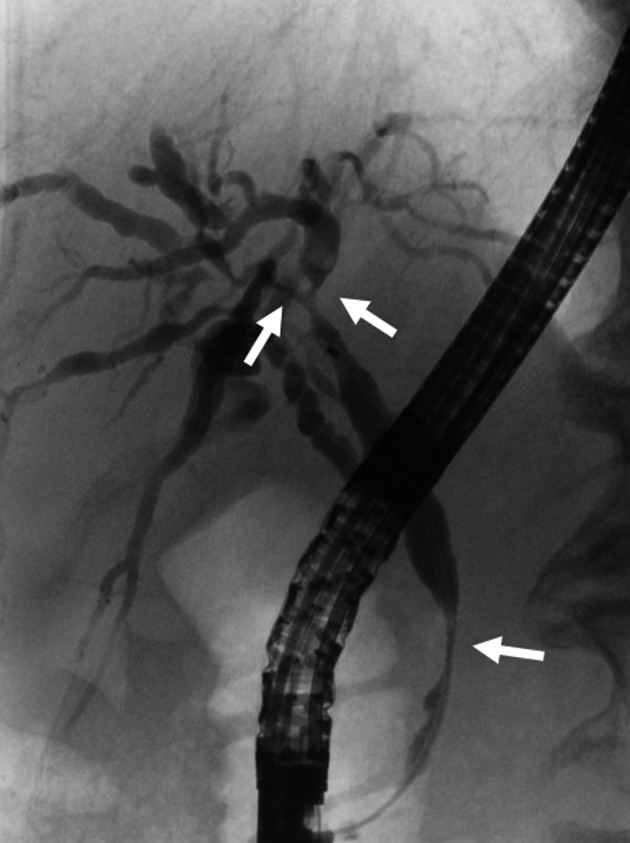
Cholangiogram of type 3 IgG4‐SC showing the hilar hepatic and lower bile duct strictures (arrows)

IgG4‐SC with localized bile duct stricture must be differentiated from cholangiocarcinoma. Cholangiography cannot be used to differentiate between the hilar hepatic strictures of type 3 (Fig. 25) or type 4 IgG4‐SC and hilar cholangiocarcinoma. The diagnostic procedures that are useful for making the differential diagnosis between types 3 and 4 IgG4‐SC and cholangiocarcinoma are endoscopic modalities such as EUS, IDUS, and bile duct cytology and/or biopsy 16, 20, 65, 98, 99 (Fig. 19). Type 1 IgG4‐SC must be differentiated from lower bile duct cancer and pancreatic cancer (Fig. 19). Diagnosis of IgG4‐SC associated with AIP is relatively easy when endoscopic retrograde pancreatography shows narrowing of the main pancreatic duct, which is characteristic of AIP, but it is very difficult to make the differential diagnosis between isolated IgG4‐SC and lower bile duct cancer 24.

Endoscopic transpapillary bile duct biopsy is performed to rule out cholangiocarcinoma, but it is difficult to obtain sufficient biliary tract tissue to evaluate for the characteristic histologic findings of IgG4‐SC (i.e. storiform fibrosis and obliterative phlebitis). A commonly used cut‐off value for IgG4‐positive plasma cell infiltration is 10 cells per HPF. Reports of the sensitivity of transpapillary bile duct biopsy as a basis for making a diagnosis of IgG4‐SC have ranged from 18 to 88% 16, 20, 100.

##### CQI‐7) How is IgG4‐SC differentially diagnosed from cholangiocarcinoma?

- We recommend determination of the serum IgG4 level, concomitant diseases, cholangiogram, bile duct wall findings of contrast‐enhanced CT or IDUS, mucosal findings of POCS, pathologic findings of biopsy or brush cytology, and clinical course for the differential diagnosis of IgG4‐SC from cholangiocarcinoma (Recommendation 1, level D).

*Comment:* The differential diagnosis of IgG4‐SC is often difficult. Cases involving types 1, 3, and 4 disease are difficult to distinguish from cholangiocarcinoma (Fig. 19). The sensitivity and specificity in the diagnosis of IgG4‐SC based on cholangiogram by endoscopic retrograde cholangiopancreatography (ERCP) are 45% and 88% 101. Diagnosis by cholangiogram using ERCP combined with CT and MRI has a sensitivity of 70–90% and specificity of 73–87% 101, 102. However, a cholangiogram is crucial for differential diagnosis of IgG4‐SC from PSC because of specific findings. Several points facilitate a differential diagnosis of IgG4‐SC based on cholangiogram findings. Some cholangiocarcinomas exhibit high serum IgG4 levels, as in IgG4‐SC; the sensitivity and specificity of IgG4 level determination for the diagnosis are 64–100% and 81–88%, respectively, with an IgG4 cut‐off level of 140 mg/dl 68, 102. A higher cut‐off level of 560 mg/dl (four times the upper normal limit) provides for more reliable differential diagnosis of IgG4‐SC from cholangiocarcinoma, with a sensitivity of 17% and specificity of 99% 69.

IgG4‐SC is often accompanied by the IgG4‐related disease AIP 1, 103. Then, the presence of concomitant IgG4‐related disease suggests the possibility of IgG4‐SC, thus warranting screening of the whole body.

Contrast‐enhanced CT is useful for differential diagnosis, and homogeneous enhancement of the bile duct walls in the arterial phase is characteristic of IgG4‐SC. By contrast, in cases involving cholangiocarcinoma, the bile duct wall is enhanced in two layers. Smoothness of the inner lumen and the outer layer is also characteristic of IgG4‐SC 88, 104, 105. An additional advantage of contrast‐enhanced CT is that it enables the detection of tumor invasion and metastatic lesions outside the bile ducts.

IDUS is useful for differential diagnosis in conjunction with ERCP. Wall thickening in the non‐stricture areas is characteristic of IgG4‐SC, and a sensitivity of 95–100%, specificity of 91%, and accuracy of 94% were reported with a wall thickness cut‐off of 0.8 mm in non‐stricture areas 1, 20. Findings of tortuous and dilated arteries in the bile duct by POCS are features of IgG4‐SC, and partially dilated arteries were detected in a case of cholangiocarcinoma 106.

Biopsy is recommended for differential diagnosis, but the sensitivity for detecting malignancy (55–72%) was low 1, 20. In addition, IgG4 staining also showed low sensitivity (18–52%) for biopsy specimens in IgG4‐SC cases 20, 100. There was a report of an increase in the rate of positive IgG4 staining to 72% with additional papillary biopsy; however, papillary biopsy is considered a supplementary method and should not be performed alone 100. Simultaneous performance of brush cytology at strictures and bile fluid cytology were shown to be favorable for improving the diagnostic utility of bile duct biopsy.

Elevation in bile fluid IgG4 levels has been reported in cases of IgG4‐SC but not PSC and cholangiocarcinoma, with sensitivity and specificity values of 100% at a cut‐off level of 113 mg/dl 107. There is a possibility monitoring of bile IgG4 levels will be useful for differential diagnosis.

Most reports regarding the differential diagnosis of IgG4‐SC and cholangiocarcinoma discussed in this section were characterized by small sample sizes and low evidence levels. A review of these reports suggests that IgG4‐SC should be distinguished from cholangiocarcinoma using a variety of methods because the diagnostic utility of each modality alone is insufficient.

##### CQI‐8) What are the useful findings for differentiation between IgG4‐SC and PSC?

- We recommend consideration of age of onset, serum IgG4 level, coexisting diseases, image findings of biliary trees, liver histology, responsiveness to steroid therapy, and clinical course to differentially diagnose IgG4‐SC from PSC (Recommendation 1, level D).

*Comment:* PSC is a chronic inflammatory liver disease with poor prognosis characterized by progressive fibrosis of intrahepatic and extrahepatic bile ducts terminally leading to liver failure via cholestatic cirrhosis 27, 108, 109. Although PSC and IgG4‐SC exhibit similar cholangiograms, these diseases recently became distinguishable with the establishment of their etiology. As the therapeutic strategies and prognoses of these diseases differ, the differential diagnosis is of great importance.

PSC and IgG4‐SC exhibit clinical differences related to (1) age of onset, (2) serum IgG4 levels, (3) coexisting diseases, (4) image findings of biliary trees, (5) liver histology, (6) responsiveness to steroid therapy, and (7) clinical course (Table 4).

**Table 4:** Differential characteristics of IgG4‐SC and PSC

	IgG4‐related sclerosing cholangitis (IgG4‐SC)	Primary sclerosing cholangitis (PSC)
Age of onset	Elderly	Two peaks (elderly and younger)
Serum IgG4 level	Elevated	Not elevated
Coexisting diseases	IgG4‐related diseases	Inflammatory bowel diseases
Image of biliary trees	Long strictures, lower bile duct strictures	Short strictures, beaded appearance
Inflammatory site of bile duct wall	Inflammation in all layers, rare epithelial damage	Severe inflammation at lumen surface, severe epithelial damage
Liver histology	Abundant IgG4‐positive plasma cell infiltration	Periductal onion‐skin fibrosis
Treatment	Steroids	UDCA, liver transplantation
Clinical course	Good	Progressive

*UDCA* ursodeoxycholic acid

Whereas IgG4‐SC primarily affects elderly patients over 60 years of age, there are two peaks in the age distribution in PSC, one in younger (20–30 years old) patients and the other in more elderly patients 30.

The serum IgG4 level is frequently elevated in IgG4‐SC but not in PSC. A multicenter survey in Japan demonstrated a diagnostic sensitivity for IgG4‐SC of 89.9% when a cut‐off value of 135 mg/dl was employed 22. In a subsequent nationwide survey, serum IgG4 levels were elevated in 83.9% of IgG4‐SC cases 110. In contrast, high serum IgG4 levels were observed in only 11.5% and 12.9% of PSC cases in these two reports, suggesting that a serum IgG4 level is a reliable biomarker for distinguishing IgG4‐SC from PSC. However, the diagnosis of IgG4‐SC should not be made solely on the basis of serum IgG4 levels, as false‐positive results are observed in approximately 10% of PSC cases.

PSC is frequently accompanied by inflammatory bowel diseases such as ulcerative colitis, with a prevalence of 60–80% in Western countries 31, 32. A Japanese nationwide survey in 2013 reported a lower incidence of inflammatory bowel disease in PSC patients of 34%. However, endoscopic examination of the colorectum is recommended in PSC patients, regardless of the absence of symptoms of inflammatory bowel disease 23, as inflammatory bowel disease is occasionally diagnosed after a definitive diagnosis of PSC 31, 32. In contrast, IgG4‐SC is associated with AIP as well as other IgG4‐related diseases such as sialadenitis and retroperitoneal fibrosis at a high prevalence 23, but it is rarely associated with inflammatory bowel disease.

Typical imaging findings of biliary trees in PSC include band‐like strictures, beaded appearance, pruned‐tree appearance, and diverticulum‐like outpouching 95, 96. Differential features on cholangiography include longer strictures in IgG4‐SC than in PSC 1, 3, 18, 96, 97. IDUS findings of irregular inner margins reflecting ductal epithelial damage and diverticulum‐like outpouching are significantly more frequent in PSC 111, 112. This is in contrast to the findings of homogenous thickening of the inner low‐echoic layer without destruction of the three‐layer structure frequently observed in IgG4‐SC. Typical POCS findings in PSC include multiple lesions involving scarring and pseudodiverticula 113, 114.

Important liver histology findings in PSC include periductal onion‐skin fibrosis and fibrous obliterative cholangitis 33, although these are not specific for PSC. For the differential diagnosis of IgG4‐SC from PSC, fibrous cholangitis is a potential finding of PSC, whereas abundant IgG4‐positive plasma cell infiltration in portal areas is suggestive of IgG4‐SC.

With regard to medical treatment, patients with IgG4‐SC are treated effectively with corticosteroids, whereas extensive evidence suggests there are no satisfactory agents for treating PSC. Although ursodeoxycholic acid is widely used in patients with PSC, guidelines in Western countries indicate that its clinical benefit is uncertain. In addition, high‐dose ursodeoxycholic acid reportedly has no beneficial effect 34, 35, 36. PSC leads to liver failure via cholestatic cirrhosis with disease duration of more than 10 years and occasionally leads to cholangiocarcinoma, suggesting poor prognosis. Hence, liver transplantation remains the only established therapy.

##### CQI‐9) Is bile duct biopsy recommended for diagnosing IgG4‐SC?

- We suggest performing bile duct biopsy for the diagnosis of IgG4‐SC (Recommendation 2, level D).
- We suggest performing bile duct biopsy for differential diagnosis from cholangiocarcinoma (Recommendation 2, level D).

*Comment:* The diagnosis of IgG4‐SC warrants bile duct biopsy to differentiate it from other diseases, particularly cholangiocarcinoma. However, definitive diagnoses of IgG4‐SC by bile duct biopsy are sporadic. The histopathology of IgG4‐SC is characterized by diffuse lymphoplasmacytic infiltration extending from the bile duct mucosa to the serous membrane, storiform fibrosis, obstructive phlebitis, and eosinophilic infiltration, whereas the histopathology of the bile duct epithelium is often normal. In other words, histopathologically, a definitive diagnosis of IgG4‐SC by bile duct biopsy, requires the collection of samples containing the bile duct stroma. Ghazale et al. reported pathologic diagnosis of IgG4‐SC in 14 of 16 patients (88%) by bile duct biopsy 16. In contrast, Kawakami et al. 100, Naitoh et al. 20, and Hirano et al. 25 reported diagnosis of IgG4‐SC by bile duct biopsy in 15 of 29 patients (52%), 3 of 17 patients (18%), and 0 of 5 patients (0%), respectively, which cannot be considered as good outcomes (Table 5). The poor ability to diagnose IgG4‐SC by bile duct biopsy may be attributed to the fact that the endoscopic bile duct samples are often small, and collecting samples containing the bile duct stroma using biopsy forceps is challenging. Although immunostaining for IgG4 is performed to more effectively use the small samples collected, satisfactory results have yet to be obtained.

**Table 5:** Usefulness of bile duct biopsy for the diagnosis of IgG4‐SC

Author	Number of cases involving bile duct biopsy	Number of cases involving histologic diagnosis	Sensitivity
Ghazale et al. 16	16	14	88%
Kawakami et al. 99	29	15	52%
Naitoh et al. 20	17	3	18%
Hirano et al. 25	5	0	0%

When we observe localized stenosis, excluding cholangiocarcinoma is necessary. Refer to CQI‐7 for differential diagnosis from cholangiocarcinoma.

##### CQI‐10) Are endoscopic biopsy samples taken from the ampulla of Vater useful for diagnosing IgG4‐SC?

- Histopathology of endoscopic biopsy samples taken from the ampulla of Vater for IgG4‐immunostaining can be useful as a supplemental tool for the diagnosis of IgG4‐SC. We suggest this technique for use in cases involving a swollen ampulla associated with pancreatic head involvement due to AIP in particular (Recommendation 2, level D).

**Figure 26:**
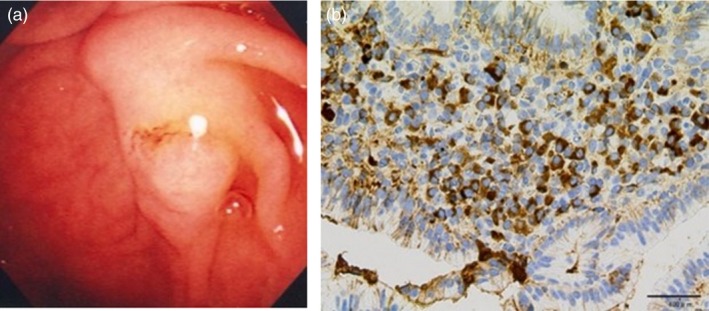
(**a**) Endoscopic image showing a swollen ampulla of Vater of a patient with IgG4‐SC. (**b**) Immunohistochemical findings of abundant infiltration of IgG4‐positive plasma cells in biopsy specimen taken from the ampulla of Vater of a patient with IgG4‐SC

*Comment:* Supplemental endoscopic biopsy histopathology using IgG4‐immunostaining could contribute to the diagnosis of IgG4‐SC in cases in which the ampulla of Vater is swollen 115 (Fig. 26a) and there is positive histopathologic evidence of infiltration (i.e. >10 IgG4‐positive plasma cells per HPF) 116 (Fig. 26b).

Features such as swollen ampulla and abundant infiltration of IgG4‐positive plasma cells are regarded as indicative of pancreatic head involvement associated with AIP rather than diagnostic of IgG4‐SC 117. There is no consensus criterion regarding endoscopic biopsy in the diagnosis of IgG4‐related disease. Additionally, endoscopic biopsy histopathology of samples taken from the ampulla of Vater is regarded as an option in the diagnosis of AIP as proposed in the international consensus diagnostic criteria 26. Supplemental endoscopic biopsy samples taken from the ampulla of Vater are useful for differential diagnosis of IgG4‐SC from PSC 118; however, endoscopic biopsy histopathology itself should not be relied on for final diagnosis of IgG4‐related diseases. One case report described isolated‐type IgG4‐related disease of the ampulla of Vater 119. Although there are no consensus diagnostic criteria available regarding the ampulla of Vater in IgG4‐related disease, it could be considered in diagnosis if there are typical endoscopic findings such as swollen ampulla of Vater associated with hooding‐fold bulging 115 and biopsy histopathologic results indicating infiltration with abundant IgG4‐positive plasma cells 116. Endoscopic findings such as a swollen ampulla of Vater and endoscopic biopsy results showing abundant IgG4‐positive plasma cell infiltration were noted in 40–80% of patients with IgG4‐SC associated with AIP 120, 121, 122. On the other hand, it would be almost impossible for the use of endoscopic biopsy histopathology to reveal stromal features of IgG4‐SC, including storiform fibrosis and obliterative phlebitis. Isolated IgG4‐SC not associated with AIP is rare 15. Furthermore, Matsubayashi reported that only 1 of 7 isolated IgG4‐SC patients presented with a swollen ampulla of Vater. That author also described the usefulness of characterizing ampulla features in diagnosing IgG4‐SC 123. Additionally, as the tissue acquisition from the affected bile ducts in IgG4‐SC patients are challenging at poor outcomes (Table 5), the feasible result taken from the ampulla, which anatomically connected to the bile duct, could be a surrogate to attest it 124. Therefore, the biopsy of the ampulla is useful. For the aforementioned reasons, ampullary features such as typical endoscopic findings indicative of pancreatic head lesions in AIP can potentially contribute to the diagnosis of IgG4‐SC; such findings would also be positive in patients with isolated‐type IgG4‐SC.

##### CQI ‐11) Is IDUS recommended for diagnosing IgG4‐SC?

- We suggest IDUS for the differential diagnosis from cholangiocarcinoma or PSC (Recommendation 2, level D).

**Figure 27:**
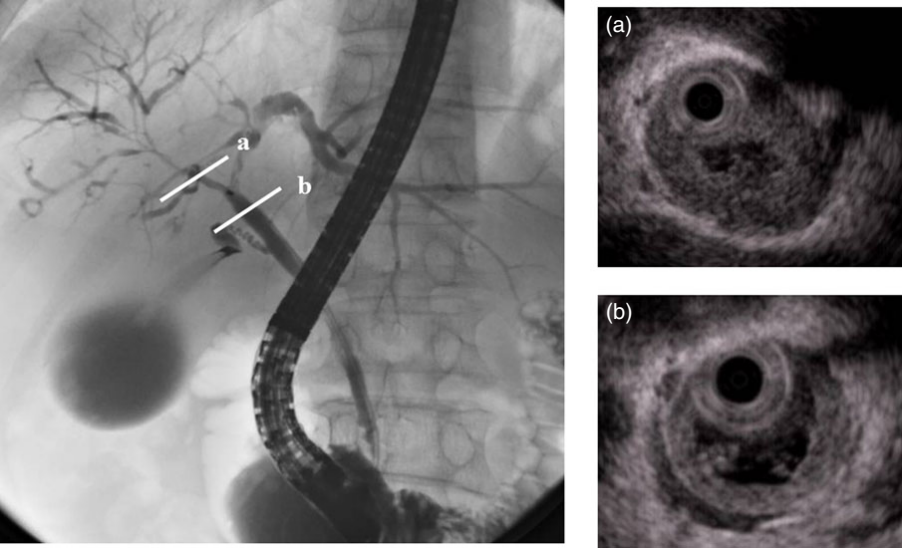
IDUS image of (**a**) biliary stricture and (**b**) non‐biliary stricture in a patient with IgG4‐SC

*Comment:* Endoscopic transpapillary IDUS is a reliable procedure to obtain high‐resolution images of the bile duct wall after ERCP. IDUS is performed by inserting a small‐caliber ultrasonic probe into the bile duct. IDUS is widely used for evaluation of bile duct stones, differential diagnosis of indeterminate biliary strictures, and superficial spread of cholangiocarcinoma. IDUS should be performed before biliary drainage, as such drainage often causes mechanical inflammation of the bile duct. IDUS findings of IgG4‐SC include circular‐symmetrical wall thickening, smooth outer and inner margins, and homogeneous internal echo in the biliary stricture (Fig. 27a) 20, 85. The most characteristic IDUS finding of IgG4‐SC is wall thickening in non‐strictures of the bile duct, which appears normal on a cholangiogram (Fig. 27b) 20. IDUS findings of IgG4‐SC reflect pathologic changes of the bile duct in which fibro‐inflammation is primarily observed in the stroma of the bile duct walls, whereas the bile duct epithelium remains intact 3.

IDUS findings in strictures and non‐strictures of the bile duct differ between IgG4‐SC and cholangiocarcinoma 3, 20, 125, 126, 127, 128, 129. IDUS findings of cholangiocarcinoma include asymmetric wall thickening, notched outer margins, rough inner margins, and homogeneous internal echo in the biliary stricture. The most characteristic IDUS finding of IgG4‐SC is wall thickening in non‐strictures of the bile duct, as mentioned above. By contrast, bile duct wall thickening is not observed in non‐stricture sites in cholangiocarcinoma due to the absence of cancer there. One study revealed that a bile duct wall thickness of 0.8 mm is the optimal cut‐off for differentiating IgG4‐SC from cholangiocarcinoma, according to receiver operating characteristic curve analysis 20. In that study 20, no cholangiocarcinoma cases exhibiting a bile duct wall thickness greater than 1 mm were noted; therefore, this cut‐off can be used to completely exclude cholangiocarcinoma from IgG4‐SC diagnoses. The most useful IDUS finding for differentiating between IgG4‐SC and cholangiocarcinoma is that wall thickness continuously spreads from the lower bile duct to the upper bile duct in most IgG4‐SC cases.

Most cases of IgG4‐SC with intrapancreatic bile duct strictures exhibit bile duct wall thickening. This IDUS finding is useful in the differential diagnosis from pancreatic cancer because intrapancreatic bile duct strictures are considered to be caused primarily by extrinsic compression due to pancreatic enlargement 25, 94. Both intrapancreatic bile duct thickening and extrinsic compression due to pancreatic enlargement influence intrapancreatic bile duct strictures. The degree of influence produced by these two factors might differ in each IgG4‐SC case. Upstream (middle and upper) bile duct thickening is often observed in IgG4‐SC but not in pancreatic cancer. An IDUS finding of upstream bile duct thickening is useful in the differential diagnosis between AIP (IgG4‐SC) and pancreatic cancer.

Typical IDUS findings of PSC include circular‐asymmetric wall thickening, irregular inner margins, unclear outer margins, diverticulum‐like outpouching, heterogeneous internal echo, and the disappearance of three layers. These findings differ from those of IgG4‐SC 111, 112. Irregular inner margins, diverticulum‐like outpouching, and the disappearance of three layers are specific findings for distinguishing IgG4‐SC from PSC according to IDUS. Diverticulum‐like outpouching is considered to be the most objective finding on ERCP 112. IDUS is more useful than ERCP for the early detection of diverticulum‐like outpouching, which is specific to PSC.

IDUS is recommended for the differential diagnosis of IgG4‐SC from cholangiocarcinoma, pancreatic cancer, or PSC, especially in patients undergoing ERCP, because IDUS can be performed after ERC.

##### CQI‐12) Is a liver biopsy recommended for diagnosing IgG4‐SC?

- Lesions of IgG4‐SC may be obtained by liver biopsy, but specific histologic features are rarely observed (Recommendation 2, level D).

*Comment:* Lesional tissues are obtained by liver biopsy in about 25% of IgG4‐SC cases 129, 130. This happens more frequently in cases with intrahepatic bile duct involvement using a 14‐G needle 129. In most cases, however, the obtained tissues reveal non‐specific histologic findings, such as lymphoplasmacytic and occasional eosinophilic infiltration in the peripheral portal regions, sometimes with periportal, intralobular, and central perivenular inflammatory cell infiltration 97, 129, 131, 132. Cholangitis similar to that in the large bile ducts, storiform fibrosis, and obliterative phlebitis are usually absent. The presence of portal‐based fibro‐inflammatory nodules in the portal tracts, resembling the findings of IgG4‐related inflammatory pseudotumors, is pathognomonic 131 but rarely observed in liver biopsies.

The standard for increased IgG4‐positive cells in the liver biopsy is >10 cells per HPF in the clinical diagnostic criteria for IgG4‐SC 3 and consensus statement on the pathology of IgG4‐related disease 48; in the latter, an IgG4/IgG‐positive cell ratio >40% also needs to be satisfied. IgG4‐positive cells in the liver biopsy are significantly more numerous in IgG4‐SC compared with PSC and cholangiocarcinomas 97, 103. In one study, >10 IgG4‐positive cells per HPF was reported in 6 of 10 IgG4‐SC cases but in none of 16 PSC cases 131. The IgG4‐positive/mononuclear cell ratio and IgG4/IgG‐positive cell ratio were reported to be significantly higher in IgG4‐SC than PSC 133. Although not previously studied with liver biopsy specimens, numerous IgG4‐positive cells may also be observed in cholangiocarcinomas; therefore, sufficient attention should be paid to incorrectly diagnose false‐negative samples of cholangiocarcinoma as IgG4‐SC based solely on IgG4‐immunostaining.

In terms of distinguishing IgG4‐SC from PSC in a liver biopsy sample, advanced fibrosis corresponding to stages 3 and 4 would be observed only in PSC and not in IgG4‐SC 14, 97. Fibrous cholangitis is significantly more frequent in PSC but can be detected in IgG4‐SC as well 97, 129, 131.

Complications, such as severe pain, vasovagal syncope, and hemorrhage, occur infrequently after a liver biopsy.

##### CQI‐13) Is POCS recommended for diagnosing IgG4‐SC?

- We suggest the use of POCS for differentiating between cholangiocarcinoma and PSC (Recommendation 2, level D).

**Figure 28:**
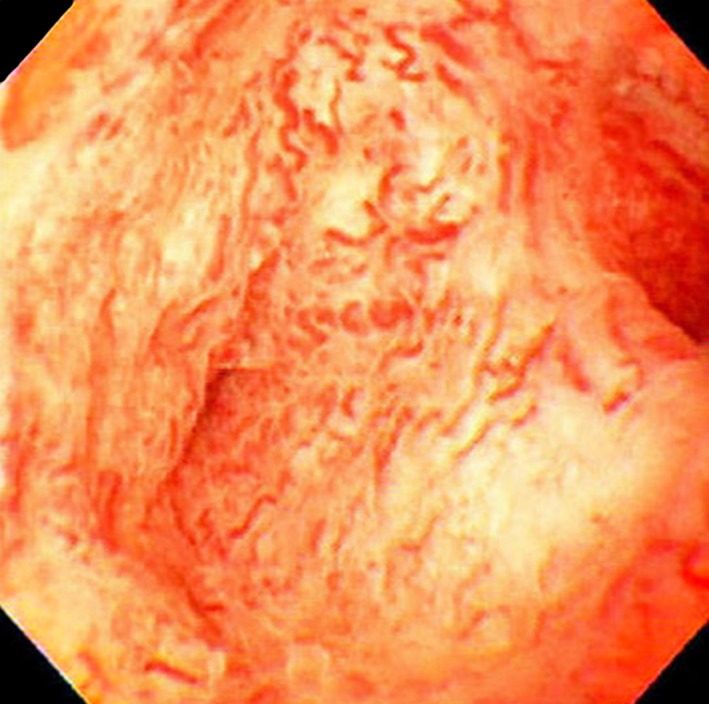
POCS image of dilated and tortuous vessels in IgG4‐SC referred from Reference 112

*Comment:* The detailed structure of the surface of the bile duct mucous membrane can be macroscopically observed using POCS. When combined with narrow‐band imaging, observation of blood vessels using POCS is useful as a diagnostic aid 113. According to a report comparing POCS images of IgG4‐SC, cholangitis, and PSC 106, a small number of cases of IgG4‐SC characteristically exhibited a high frequency of dilated and tortuous vessels, and in many instances, there was no fibrous scar tissue (Fig. 28). In IgG4‐SC, inflammation is primarily submucosal 15; therefore, it is said that the appearance of congestion in the mucosal veins, where there is relatively little inflammation, can be perceived macroscopically. By contrast, in PSC, blood vessel distribution is poor, and scarring with pseudodiverticulum‐like changes is often seen. In cholangiocarcinoma, irregular mucosal changes and large new blood vessels (particularly enlarged vessels) are often observed. However, differentiation based on POCS images alone can be difficult. In the diagnosis of cholangiocarcinoma, when bile duct biopsy under fluoroscopic guidance is difficult, specimens can be collected with greater reliability by POCS biopsy.

##### CQI‐14) Are steroid trials recommended for diagnosing IgG4‐SC?

- We suggest that steroid trials should be carried out only by experts familiar with AIP and IgG4‐SC or in patients with diagnostically challenging cases of IgG4‐SC without AIP or other organ involvement only after exclusion of cholangiocarcinoma (Recommendation 2, level D).
- The effectiveness of steroid trials for diagnosing IgG4‐SC should be assessed by confirming resolution of findings on bile duct images obtained using ERCP/MRCP after 1 or 2 weeks of steroid administration at a dose of 0.4–0.6 mg/kg. In the absence of amelioration, we suggest re‐evaluation, including resection of cases suspicious for cancer (Recommendation 2, level D).

*Comment:* There are no reports of randomized controlled steroid trials for the diagnosis for IgG4‐SC. Only one paper regarding AIP and a few case reports have been published 134, 135. A steroid trial conducted for differential diagnosis of AIP and pancreatic cancer stated that steroid trials should be carried out only by pancreatobiliary subspecialists after the exclusion of potential malignancy 26. This would also apply to the use of steroid trials in the diagnosis of IgG4‐SC, which should be attempted only after excluding biliary malignancy.

Steroid trials would be appropriate for use by pancreatobiliary subspecialists in diagnostically challenging cases mimicking PSC and/or cholangiocarcinoma. Almost all cases of IgG4‐SC are associated with AIP, and cases that are not associated with AIP are often accompanied by involvement of other organs. These diagnostic clues could facilitate a correct diagnosis of IgG4‐SC without evaluation of histopathologic evidence in bile duct samples 15. However, a steroid trial would be acceptable in diagnostically challenging patients with IgG4‐SC with a slight relationship with AIP and/or patients with isolated‐type IgG4‐SC without other organ involvement. Steroid trials might also be appropriate in patients with elevated serum IgG4 levels who present with typical cholangiogram results indicative of IgG4‐SC 18. The effectiveness of steroid trials for diagnosing IgG4‐SC should be assessed by confirming resolution of findings on bile duct images obtained using ERCP/MRCP after 1 or 2 weeks of steroid administration at a dose of 0.4–0.6 mg/kg 16, 136. The Mayo Clinic group 16 and Iwasaki et al. 136 validated the use of steroid trials for diagnosing IgG4‐SC. The efficacy of steroid treatment should be evaluated within a week or so using serum data, and CT and MRCP or ERCP imaging findings should be assessed 1 to 2 weeks after the trial. In the absence of amelioration, re‐evaluation, including resection of cases suspicious for cancer, should be proposed.

#### Treatment

##### CQII‐1) What are the recommendations for steroid therapy in IgG4‐SC patients?

- We recommend steroid therapy for almost all IgG4‐SC patients. Immediate steroid therapy should be considered for IgG4‐SC patients with obstructive jaundice, acute cholangitis, and symptomatic extrabiliary IgG4‐related diseases (Recommendation 1, level D).

*Comment*: Steroid therapy is recommended as the standard treatment for IgG4‐related disease 2, 137, 138. According to a nationwide survey in Japan, about 88% of IgG4‐SC patients receive steroid therapy 19. The steroid‐treated IgG4‐SC remission rate is 90% 19.

According to the Japanese consensus guidelines for AIP, the indications for steroid therapy in AIP are the presence of symptoms (e.g. obstructive jaundice, abdominal pain, and back pain) and the presence of symptomatic extrapancreatic lesions 139. Furthermore, based on the international consensus guidance statement on the management and treatment of IgG4‐related disease, proximal biliary strictures as well as associated symptomatic IgG4‐related diseases, including aortitis, retroperitoneal fibrosis, tubulointerstitial nephritis, pachymeningitis, pericarditis, and diffuse pancreatic enlargement, have been reported as indications for urgent steroid therapy, which may include combination therapy consisting of higher steroid doses and other mechanical interventions, such as biliary stenting 138. Thus, it is recommended that almost all IgG4‐SC patients receive steroid therapy. Immediate steroid therapy should be considered in IgG4‐SC patients with obstructive jaundice, acute cholangitis, and symptomatic extrabiliary IgG4‐related diseases 138.

It was reported that jaundice in some IgG4‐SC cases without acute cholangitis can be safely treated and effectively managed with steroid therapy alone, without any need for biliary stenting 140. However, biliary drainage should be considered in IgG4‐SC cases with acute cholangitis diagnosed based on the 2013 Tokyo Guidelines 126, 141, 142.

Biliary stricture relapse has been reported in IgG4‐SC patients during steroid tapering or after withdrawal of steroid treatment, and development of new biliary strictures has been reported in untreated IgG4‐SC patients 16, 40, 143, 144. Relapse rates reportedly range from 16% 16 to 53% 144. Bile duct strictures reportedly improve to varying degrees after steroid treatment, but mild bile duct strictures can persist in some IgG4‐SC patients 16, 40. Consequently, steroid treatment is also recommended for asymptomatic IgG4‐SC patients with elevated serum hepatobiliary enzyme levels. Differential diagnosis between cholangiocarcinoma and pancreatic cancer should be made before starting steroid therapy in IgG4‐SC patients.

##### CQII‐2) Is biliary drainage recommended before initiating steroid therapy for IgG4‐SC?

- We suggest performing biliary drainage in cases involving obstructive jaundice due to biliary stenosis (Recommendation 2, level D).
- Steroid therapy can be initiated without biliary drainage in cases involving mild jaundice without cholangitis in patients for whom the diagnosis is definite and pathologic approaches to the biliary stenosis are unnecessary (Recommendation 2, level D).

*Comment*: In IgG4‐SC, it is important to differentiate bile duct invasion by pancreatic cancer or lower bile duct cancer in cases involving stenosis of the lower bile duct and to differentiate from hilar cholangiocarcinoma in cases involving stenosis of the upper or hilar bile duct. Regarding differential diagnosis, ERCP and related procedures play an important role. Many IgG4‐SC patients undergo ERCP, IDUS, transpapillary bile duct biopsy, and/or bile cytology with/without brushing, with ultimate insertion of an endoscopic nasobiliary drainage tube or plastic stent to relieve cholestasis and prevent cholangitis in a single session 138, 142, 145. According to one Japanese study 146 and an international survey 147, biliary drainage before starting steroids were underwent in 77% and 71% of AIP patients who presented with obstructive jaundice, respectively.

On the other hand, in a study of 15 AIP patients, Bi et al. of the Mayo Clinic 140 reported that obstructive jaundice was safely and effectively treated with steroids alone without biliary stenting. Follow‐up serum liver tests at a mean of 4 days (range 1–14 days) after starting steroids showed rapid reduction in all measures: 54.9% decrease in aspartate aminotransferase (AST), 51.6% decrease in alanine aminotransferase (ALT), 33% decrease in alkaline phosphatase (ALP), and 47.2% decrease in total bilirubin (TB). By 15 to 45 days after steroid treatment, all patients had normal AST, three patients had ALT more than 1.5 × ULN (upper limit of normal), one patient had ALP more than 1.5 × ULN, and only one patient had TB more than 1.5 × ULN. No patients showed any infectious complications such as cholangitis during steroid treatment. Therefore, steroid therapy alone in a closely monitored setting under the guidance of an experienced pancreatologist is recommended for AIP patients with obstructive jaundice whose diagnosis is certain, thereby avoiding ERCP and its potential complications such as pancreatitis.

Iwasaki et al. 136 reported 75%, 89%, and 83% decreases in serum levels of ALT, ALP, and TB, respectively, around 5 days after starting steroids in 19 patients with IgG4‐SC who were treated with steroids without biliary drainage, and the levels were halved in 63%, 89%, and 67% around 10 days after starting steroids. Bile duct stenosis improved in all patients except one on ERC performed 1–4 weeks after starting steroids.

The above data suggest that as IgG4‐SC improves rapidly with steroids, steroid therapy can be initiated without ERC or biliary drainage in cases involving mild jaundice without cholangitis in patients for whom the diagnosis is definite and pathologic approaches to the biliary stenosis are unnecessary, such as in cases of stenosis of the lower bile duct associated with elevated serum IgG4 levels and diffuse enlargement of the pancreas 94, 148.

##### CQII‐3) How is steroid therapy for IgG4‐SC performed?

- We suggest oral prednisolone at 0.6 mg/kg/day (usual dose: 30–40 mg/day) for 2–4 weeks for initial remission‐induction therapy (Recommendation 2, level D).
- We suggest that the dose of prednisolone is reduced by 5 mg every 1–2 weeks after induction of remission, while confirming the response to steroid therapy by laboratory tests and imaging studies (e.g. ultrasonography, CT, or MRCP), and then gradually tapered further to the maintenance level by 2–3 months (Recommendation 2, level D).
- Although the recommended maintenance dose is 5 mg/day, adjusting the dose within the range of approximately 5 mg/day based on the patient's response is suggested (Recommendation 2, level D).
- After remission has been maintained for 3 years, it is possible to consider further reducing or discontinuing steroid therapy, but we suggest that this should be done carefully because there is a high risk of relapse (Recommendation 2, level C).
- We recommend periodic assessment of symptoms, analysis of objective findings such as jaundice, and biochemistry tests, serum IgG/IgG4 levels, and imaging findings after the initiation of steroid therapy as well as after discontinuation of therapy. Care should be taken to identify relapse, exclude malignancy, and control adverse reactions to steroid therapy while managing the clinical course of these patients (Recommendation 1, level D).

*Comment:*

1) Initial remission induction

There have been few studies on steroid therapy for IgG4‐SC. Steroid therapy is indicated for patients who have AIP with symptoms, jaundice, and bile duct involvement. The standard treatment schedule recommended by the Japanese clinical guidelines for AIP 139 and the international consensus diagnostic criteria 148 is oral prednisolone (0.6 mg/kg/day) for 2–4 weeks as initial remission‐induction therapy, with subsequent tapering by 5 mg/day every 1–2 weeks and reduction to the maintenance dose by 2–3 months after the start of treatment, observing the patient's response throughout 146, 149. The Mayo Clinic also proposed a treatment schedule that starts with 4 weeks of oral prednisone at 40 mg/day, followed by tapering at 5 mg/week until discontinuation at 11 weeks 16, 150. In a Japanese multicenter study, biliary drainage was performed in 242/314 patients who had obstructive jaundice among 563 patients with AIP in order to compare a starting dose of 40 mg/day or 30 mg/day of oral prednisolone in 459 patients treated with steroids, but there was no difference of the time to remission or the relapse rate 146. Although there are reports of an observable effect even at low steroid doses (≤20 mg/day) in diabetic patients who are at risk of exacerbation of diabetes by steroid therapy 19, 151, patient selection bias cannot be ruled out in retrospective studies. It was also reported that steroid pulse therapy is more effective for improving early bile duct lesions compared with oral steroids when it is desirable to obtain a response in a short period 152, 153.

2) Dose reduction after remission induction

After induction of remission, the response to steroids is confirmed by laboratory tests and imaging studies. Steroid therapy is continued, with the dose gradually being tapered if other diseases are excluded, including cholangiocarcinoma, pancreatic cancer, and PSC. If improvement is not achieved by steroid therapy and the possibility of another disease is considered to be high, it is necessary to promptly review the diagnosis. After induction of remission with steroid therapy, the standard method is to reduce the dose of prednisolone by 5 mg every 1–2 weeks until the maintenance dose (5–10 mg/day) is finally reached at 2–3 months after starting treatment. Since it has been reported that there is a risk of relapse of SC, the steroid dose should be reduced while regularly checking for recurrence by monitoring symptoms and signs, biochemistry data, IgG/IgG4 levels, and imaging findings (e.g. ultrasonography, CT, MRCP, or ERCP). After prednisolone is reduced to 15 mg/day, the dose is reduced further at longer intervals in some cases 146. While pancreatic enlargement associated with AIP almost always improves after remission is achieved by steroid therapy, bile duct stenosis persists in 58% of patients with persistent elevation of IgG4 versus 27% of patients with normal IgG4 levels 146.

3) Maintenance therapy

A randomized controlled trial of steroid maintenance therapy was performed in 49 Japanese patients with AIP after induction of remission to compare maintenance therapy (5–7.5 mg/day) between a group treated for 3 years and a group in which therapy was discontinued at 26 weeks. It was found that the relapse rate up to 3 years was 23.3% in the former group versus 57.9% in the latter, and the long‐term maintenance therapy group had a significantly lower risk of relapse, with no serious steroid‐related adverse events 154. In a retrospective multicenter study of 510 patients with AIP 155, the relapse rate achieved with standard steroid therapy for AIP 139 was 10% at 1 year, 11% at 2 years, 25.8% at 3 years, 30.9% at 4 years, and 35.1% at 5 years, finally reaching a plateau at 43% after 7 years. When maintenance therapy was performed with a steroid dose of 2.5–10 mg/day, the relapse rate was significantly lower in the group that received ongoing maintenance therapy than in the group with withdrawal of therapy (30.3% vs. 45.2%). Also, when administration of oral prednisolone at various doses (0, 2.5, 5, 7.5, or 10 mg/day) was compared, the risk of relapse was the lowest at 5 mg/day (26.1%), and there were no significant differences between 5 mg, 7.5 mg, and 10 mg/day. Discontinuing steroid therapy in the short term has been the general policy in other countries. However, a retrospective study of 30 patients with SC performed at the Mayo Clinic showed that starting oral prednisone at 40 mg/day with tapering by 5 mg/week until discontinuation at 11 weeks resulted in a recurrence rate of 53% after a median of 3 months and 71% within 6 months 16. In a prospective study of IgG4‐SC performed in the United Kingdom, oral prednisolone was initiated at 30 mg/day for 2 weeks and then reduced by 5 mg every 2 weeks, resulting in a remission rate of 82%. However, 18% of the patients had no response to steroid therapy, and 35% developed recurrence after a median of 4 months 135. From these results, the consensus in Japan is that continuing long‐term maintenance therapy at a dose of approximately 5 mg/day for 3 years is desirable to prevent recurrence 139, 146, but there is no agreement regarding discontinuation of steroid therapy or further dose reduction after 3 years. Hirano et al. 156 performed a prospective trial in 21 patients with AIP who discontinued maintenance therapy after 3 years, and they found relapse in 48% of the patients during follow‐up for 43 months. Based on these results, they recommended that long‐term maintenance therapy should be continued beyond 3 years. A Japanese multicenter study of adverse events associated with steroid therapy reported an area under the curve of 0.717 when the cut‐off value for the total steroid dose was set at 6,405 mg 155. To avoid adverse reactions caused by long‐term administration of corticosteroids, the standard recommendation is to consider continuation of therapy, dose reduction, or discontinuation after 3 years, as the risk of infection increases with long‐term administration at 10 mg/day, and the risk of steroid‐associated osteoporosis is significantly increased when the total steroid dose reaches or exceeds 6,405 mg. In IgG4‐SC patients, we should carefully consider steroid dose reduction, maintenance therapy, and the duration of treatment for those with SC, as it has a high risk of relapse.

##### CQII‐4) How should relapse be treated?

- We recommend both re‐administration and increasing the dose of steroids for treating relapse in IgG4‐SC patients (Recommendation 1, level D).
- Immunomodulatory drugs and rituximab are useful in some relapse cases. However, treatment with these drugs is not covered by the medical insurance system in Japan (level C).

*Comment:* Relapse of IgG4‐SC is generally defined as a reappearance of symptoms after remission (during maintenance steroid therapy or after steroid withdrawal), with the development or aggravation of bile duct strictures, and/or other organ involvement (e.g. AIP, IgG4‐related dacryoadenitis/sialadenitis, IgG4‐related retroperitoneal fibrosis) with abnormalities on imaging, and/or elevation of serum IgG4 levels 157, 158. Re‐elevation of serum IgG4 levels alone without symptoms or with the presence of bile duct strictures is not considered to be relapse 157, 158.

Disease relapse occurs in 30–57% of IgG4‐SC patients either during maintenance steroid therapy or after the discontinuation of steroids, particularly in the first couple of years 58, 147. Relapse rates after steroid therapy are similar to those reported after surgery 16. In a retrospective cohort study of 507 patients with IgG4‐SC in Japan, relapse with restenosis of the bile ducts was noted in 104 patients (19%), and the cumulative rate of relapse was 1.6%, 7.6%, and 16.5% at 1, 3, and 5 years after diagnosis, respectively 19. Known risk factors predictive of a relapse include high serum IgG4 level at diagnosis, the presence of proximal extrahepatic/intrahepatic or multiple bile duct strictures, and thicker bile duct walls during the initial attack 16, 147, 157, 159.

Both re‐administration and increasing the dose of steroids have been shown to be effective in treating relapse patients 139, 157. In Western countries, in addition to the re‐administration of steroids, immunomodulatory drugs such as azathioprine, 6‐mercaptopurine, mycophenolate mofetil, and methotrexate have been administered as steroid‐sparing agents for treating relapse in IgG4‐SC patients 16, 58, 135, 141, 157. However, the benefits of the additional immunomodulators in reducing time to further relapse are uncertain, and as these drugs are associated with serious side‐effects, their use should be considered with caution 157.

Rituximab, a monoclonal anti‐CD20 antibody leading to B‐cell depletion, is reportedly effective for treatment of IgG4‐SC resistant to steroid and immunomodulator therapy. According to the limited data available, disease response is obtained in 80–90% of IgG4‐SC patients, including many with difficult‐to‐treat disease 157, 160, 161, 162.

In international consensus statements for the treatment of AIP, immunomodulatory drugs or rituximab is recommended for relapse AIP as steroid‐sparing agents. However, treatment with these drugs for IgG4‐related diseases including IgG4‐SC is not covered by the medical insurance system in Japan 148.

## Conflict of interest

None declared.

## Appendix 1

The members of the guideline committee who created and evaluated these guidelines are listed below.

### Creation committee

Chair: Terumi Kamisawa (Department of Internal Medicine, Tokyo Metropolitan, Komagome Hospital, Tokyo, Japan)

Vice‐Chair: Takahiro Nakazawa (Department of Gastroenterology, Japanese Red Cross Nagoya Daini Hospital, Nagoya Japan)

Members: Susumu Tazuma (Department of General Internal Medicine, Hiroshima University Graduate School of Biomedical & Health Science, Hiroshima, Japan), Yoh Zen (Department of Diagnostic Pathology, Kobe University, Kobe, Japan), Atsushi Tanaka (Department of Medicine, Teikyo University School of Medicine, Tokyo, Japan), Hirotaka Ohara (Department of Community‐Based Medical Education, Nagoya City University Graduate School of Medical Sciences, Nagoya, Japan), Takashi Muraki (Department of Medicine, Gastroenterology, Shinshu University, Matsumoto, Nagano, Japan), Kazuo Inui(Department of Gastroenterology, Second Teaching Hospital, Fujita Health University, Nagoya, Japan), Dai Inoue (Department of Radiology, Kanazawa University Graduate School of Medical Sciences, Kanazawa, Japan), Takayoshi Nishino (Department of Gastroenterology, Tokyo Womens’ Medical University Yachiyo medical center, Yachiyo, Japan), Itaru Naitoh (Department of Gastroenterology and Metabolism, Nagoya City University Graduate School of Medical Sciences, Nagoya, Japan), Takao Itoi (Department of Gastroenterology and Hepatology, Tokyo Medical University, Tokyo, Japan), Kenji Notohara (Department of Anatomic Pathology, Kurashiki Central Hospital, Kurashiki, Japan), Atsushi Kanno (Division of Gastroenterology, Tohoku University Graduate School of Medicine, Sendai, Japan), Kensuke Kubota (Department of Endoscopy, Yokohama City University Hospital, Yokohama, Japan), Kenji Hirano (Department of Gastroenterology, Tokyo Takanawa Hospital, Tokyo, Japan), Hiroyuki Isayama (Department of Gastroenterology, Graduate School of Medicine, Juntendo University), Kyoko Shimizu (Department of Gastroenterology, Tokyo Womens’ Medical University, Tokyo, Japan, Toshio Tsuyuguchi (Endoscopy Center, Chiba University Hospital, Chiba, Japan).

### Expert panellist committee

Chair: Tooru Shimosegawa (Division of Gastroenterology, South‐Miyagi Medical Center, Ohgawara, Japan).

Members: Terumi Kamisawa (Department of Internal Medicine, Tokyo Metropolitan, Komagome Hospital, Tokyo, Japan), Takahiro Nakazawa (Department of Gastroenterology, Japanese Red Cross Nagoya Daini Hospital, Nagoya Japan),　 Susumu Tazuma (Department of General Internal Medicine, Hiroshima University Graduate School of Biomedical & Health Science, Hiroshima, Japan), Atsushi Tanaka (Department of Medicine, Teikyo University School of Medicine, Tokyo, Japan), Hirotaka Ohara (Department of Community‐Based Medical Education, Nagoya City University Graduate School of Medical Sciences, Nagoya, Japan), Kazuo Inui (Department of Gastroenterology, Second Teaching Hospital, Fujita Health University, Nagoya, Japan), Shigeyuki Kawa (Department of Internal Medicine, Matsumoto Dental University), Kensuke Kubota (Department of Endoscopy, Yokohama City University Hospital, Yokohama, Japan), Kenji Notohara (Department of Anatomic Pathology, Kurashiki Central Hospital, Kurashiki, Japan).

### Evaluating committee

Chair: Tsutomu Chiba (Kansai Electric Power Hospital, Osaka, Japan).

Members: Kazuichi Okazaki (The Third Department of Internal Medicine, Division of Gastroenterology and Hepatology, Kansai Medical University, Moriguchi, Japan), Hajime Takikawa (Department of Medicine, Teikyo University School of Medicine, Tokyo, Japan), Wataru Kimura (Departments of Gastroenterology and Gastroenterological, General, Breast, and Thyroid Surgery, Yamagata University Faculty of Medicine, Yamagata, Japan), Michiaki Unno (Division of Hepato‐Biliary Pancreatic Surgery, Tohoku University Graduate School, of Medicine, Sendai, Japan), Masahiro Yoshida (Department of Hepato‐Biliary Pancreatic and Gastrointestinal Surgery, International University of Health and Welfare, Ichikawa Hospital, Ichikawa, Japan).
